# Human movement patterns of farmers and forest workers from the Thailand-Myanmar border

**DOI:** 10.12688/wellcomeopenres.16784.2

**Published:** 2023-10-02

**Authors:** Sai Thein Than Tun, Myo Chit Min, Ricardo Aguas, Kimberly Fornace, Gay Nay Htoo, Lisa J. White, Daniel M. Parker

**Affiliations:** 1 Mahidol-Oxford Tropical Medicine Research Unit, Faculty of Tropical Medicine, Mahidol University, Bangkok, Thailand; 2 Centre for Tropical Medicine and Global Health, Nuffield Department of Medicine, University of Oxford, Oxford, UK; 3 Shoklo Malaria Research Unit, Mahidol Oxford Tropical Medicine Research Unit, Faculty of Tropical Medicine, Mahidol University, Mae Sot, Thailand; 4 Centre for Climate Change and Planetary Health, London School of Hygiene & Tropical Medicine, London, UK; 5 Faculty of Infectious and Tropical Diseases, London School of Hygiene & Tropical Medicine, London, UK; 6 Big Data Institute, Li Ka Shing Centre for Health Information and Discovery, Nuffield Department of Medicine, University of Oxford, Oxford, UK; 7 Department of Population Health and Disease Prevention, University of California, Irvine, CA, 92697, USA; 8 Epidemiology and Biostatistics, University of California, Irvine, CA, 92697, USA

**Keywords:** human movement, human ecology, disease ecology, infectious disease epidemiology, Thailand-Myanmar border, forests, farms

## Abstract

**Background**: Human travel patterns play an important role in infectious disease epidemiology and ecology. Movement into geographic spaces with high transmission can lead to increased risk of acquiring infections. Pathogens can also be distributed across the landscape via human travel. Most fine scale studies of human travel patterns have been done in urban settings in wealthy nations. Research into human travel patterns in rural areas of low- and middle-income nations are useful for understanding the human components of epidemiological systems for malaria or other diseases of the rural poor. The goal of this research was to assess the feasibility of using GPS loggers to empirically measure human travel patterns in this setting, as well as to quantify differing travel patterns by age, gender, and seasonality among study participants.

**Methods**: In this pilot study we recruited 50 rural villagers from along the Myanmar-Thailand border to carry GPS loggers for the duration of a year. The GPS loggers were programmed to take a time-stamped reading every 30 minutes. We calculated daily movement ranges and multi-day trips by age and gender. We incorporated remote sensing data to assess patterns of days and nights spent in forested or farm areas, also by age and gender.

**Results**: Our study showed that it is feasible to use GPS devices to measure travel patterns, though we had difficulty recruiting women and management of the project was relatively intensive. We found that older adults traveled farther distances than younger adults and adult males spent more nights in farms or forests.

**Conclusion**: The results of this study suggest that further work along these lines would be feasible in this region. Furthermore, the results from this study are useful for individual-based models of disease transmission and land use.

## Introduction

Human movement or travel is important with regard to infectious disease epidemiology and ecology ^1, 2^. Infectious diseases are heterogeneously distributed across landscapes. Individuals may be exposed to greater risk of acquiring infection if they move through transmission hotspots. Infectious individuals who travel may disperse pathogens across the landscape. Healthcare facilities are also heterogeneously distributed across landscapes, with ramifications for individual, household, and community access to diagnosis and treatment. Generally speaking, individuals who must travel long distances or through difficult terrain in order to seek diagnosis or treatment are less likely to receive adequate treatment ^3– 5^.

As early as the 1950s, the human movement was recognized as one of the most important factors for disease elimination and eradication ^6^. A growing number of research projects, some focused on health, are recording human movement patterns ^7– 12^. There have been attempts to map human movement in the rural Thailand border areas to delineate and intervene the risks of malaria ^13– 15^. These projects can be broadly divided into those that are based on questionnaires/interviews and those that are based on empirical measurements (GPS devices, mobile phones, tweets, etc.) All approaches have strengths and weaknesses ^16^. Interview/questionnaire-based approaches are prone to recollection bias and some movements may be unreported because of their nature (for example, if movements are made for illegal purposes or to places that participants don’t want to discuss/report). 

Mobile phone records provide a source of movement information across broad swaths of many populations ^17^. However, the movement data are limited to the resolution of mobile tower density, and mobile phone towers are not evenly distributed across landscapes (they tend to be clustered in urban settings). There is bias in who owns and uses mobile phones as well ^18^ and mobile phone records will not allow for fine-scale mapping of the routes travelled in between locations ^19^.

Wearable GPS devices offer extremely detailed data, but are labor intensive and dependent on volunteer cohort members. However, as the devices have become more compact (increasing wearability) and have become more affordable, their use is increasingly common ^10, 20– 26^.

The main goals of this pilot project were to: I.) assess the feasibility of using GPS loggers to track human movement patterns among people living on the Thailand-Myanmar border, and II.) measure human movement patterns, including how they vary seasonally, among a cohort of participants. The results of this work have implications for further research in this region with regard to targeted public health interventions, normal travel patterns and related exposure to different environments, for individual risk of infection by various diseases (e.g. SARS-CoV-2, malaria, melioidosis), and with regard to human disease ecology. The resulting data can also be useful for calibrating human movement patterns of individuals in an individual based modelling system.

## Methods

### Context of the study area

The study area is on the Thailand-Myanmar border. Participants were recruited from clinics that serve rural, mostly underdeveloped, and low population density communities. Most participants were of the Karen ethnic group. Villages were made up of a few dozen of mostly multigeneration families living in stilt houses made of wood and thatched roof. Villages didn’t normally have schools, clinics, or sanatory toilets. The houses are normally located along the main dirt road of the village. The dirt roads then continued to connect to other villages and small towns through a hilly and rugged terrain with occasional watersheds and river basins which made traversing difficult, especially during the rainy season.

Villagers made their living mostly though agriculture, but they have to undertake various types of jobs throughout the year for their subsistence. They developed land in and around their villages into farms to cultivate rice and vegetables. They farm poultry and pigs under their stilt houses. Some villagers go into the forests for hunting, or for foraging wood, and to collect wild edibles. They would go to the farms and forests overnight occasionally, and sleep without much protection from mosquitos and insects. We focused on farms and forests as places of interest in this study since apart from their homes, farms and forests might be the places the villagers spend significant amount of their time while being vulnerable to infectious diseases such as malaria.

### Data

The study period began in March 2017 and ended in February 2018 and aimed to recruit 50 participants for a one-year duration of time ( ClinicalTrials.gov Identifier: NCT03087214, March 22 2017). The study size was purposive as this was an exploratory pilot study and no power analysis was used. Prior to the study beginning we held community engagement meetings with community elders in the Tak Province Community Ethics Advisory board (T-CAB) to explain the project. The study locations were selected because of community enthusiasm to participate and operational feasibility. Participants were recruited from 10 villages (the lowest level of administrative division in Myanmar) near two clinics on the Thailand-Myanmar border: Wang Pah and Maw Ker Tai Clinics ( *Extended data*: Figure S1 ^27^). These clinics primarily serve migrant and cross border populations and have connections to village health workers in nearby villages. We reached out to village health workers in the nearby villages to explain the project and to ask if they could help us recruit participants from their respective villages. Participants were recruited and interviewed at the respective clinics. There were no house visits and all data were collected at the clinics. 

The study targeted individuals who were 18 years of age and above from the Karen or Burmese ethnic groups, who stated that they would be able to keep track of the GPS device, who were capable of walking outside of village boundaries at recruitment, and who were willing to provide written consent to the study. As incentives, participants were provided with a waterproof handbag at the beginning of the study, a headlamp in the middle of the study, and a jacket at the end of the study. The total cost of incentives per person was less than 10 GBP.

Upon recruitment, the age and gender of each participant was recorded following receipt of written informed consent (in Karen language). Participants were asked to carry i-gotU GT-600 (46x41.5x14mm) mobile GPS devices for the study. They have a reported average location error of less than 10 meters ^28^. They were programmed to take a reading every 30 minutes. The devices are equipped with motion sensors and were set to go into a dormant mode if they sat still for longer than one hour, and to resume taking GPS readings upon detection of movement. Devices were also set to take readings at one-minute intervals if the device was moving ≥ 15km/hour (travelling by vehicle rather than walking).

Field managers (one per clinic, MCM and GNH) met with study participants each month. During these meetings participants were questioned about their continued willingness to participate in the study by the field managers, their general movement patterns during the previous month and with regard to any illnesses. A newly charged GPS device was given to each participant (two GPS devices were devoted to each study participant, total of 100 devices used) during each of these monthly meetings and the GPS logger that had been carried during the previous month was collected for re-charging. The GPS device batteries last roughly 1 to 1 ½ months.

The data were transferred to a computer and stored in an encrypted folder with a unique code for each person to maintain security and anonymity. Separate data files were combined to obtain aggregated, longitudinal data for each participant. QGIS version 3.4.9 was used to generate study location maps and to visually explore the raw data. R statistical software version 4.0.3 was used for the data processing and analysis ^29, 30^, using the “sp”, “rgdal”, “raster”, “proj4”, “reshape” and “ggplot2” R packages ^31– 36^. GPS coordinates, which were originally recorded in 1984 World Geographical Coordinate System (WGS 84), were projected to UTM zone 47N to perform geographical calculations.

Land cover types (farms and forests) were classified manually (by hand) using satellite imagery from Google Earth (version 7.3.3.7786). Farms could be differentiated from forests by presence of human intervention on the vegetation cover e.g., vegetation cover in the farms were in more or less neatly arranged rows/columns. While formal ground truthing was not done after categorization, the locations of farms and forests do correspond to our experiences on the ground in these villages. 

### Analysis

Our analyses focused on quantifying daily movement ranges, multi-day trips, and time spent in farm or forest areas across population strata.

The last GPS point of the day between 6pm to 12 midnight was considered to be the location where an individual spent the night. The median center of these points was assumed to be the individual’s home location. A buffer with 266 meters radius (which is the standard distance deviation of accuracy of the GPS device placed inside a bag inside a house; details in *Extended data*: Figure S2 ^27^) was created around each home to create a polygon (a GIS object with a series of x and y coordinate pairs that represents an enclosed area on a map) for home area. Polygons for the farms and forests were manually classified using satellite imagery from Google Earth.

As a proxy for how far people move each day, we calculated the maximum daily Euclidian distance, which is the furthest Euclidian distance a person was away from the location he or she slept the previous night. Multiday trips away from home were identified when the minimal daily Euclidian distances were more than 266 meters from the individual’s home location consecutively for two or more days.

The Wilcoxon rank-sum test was used to compare the distributions of maximum daily Euclidian distances. A negative-binomial generalized linear mixed-effects model was used to investigate potential associations between the total number of nights spent in the farms or forests (response variables) and other characteristics such as age group, gender, and season (exploratory variables). As there were multiple observations per individual (for each time step), a random intercept was used for individuals.

Utilization of places (home, farms or forests) for each person was estimated using two different approaches. The first method was by checking whether more than two temporally-consecutive GPS points of a person fall within a polygon designated for the person’s home (for this particular measurement, the polygon is a circle of radius 266 meters around the person’s home location), farms, or forests on each day. This is equivalent to checking if a person spent at least an hour within the same polygon. For each participant, the number of days spending in each category of place (home, farm, forest) was divided by the total number of days participated during the study period to obtain the proportion of being at the respective places.

The second method estimated the utilization of places by a biased random bridge (BRB) technique ^37, 38^. Unlike prior methods for estimation of utilization of places such as location-based kernel density estimations (LKDE), BRB takes the activity time between successive relocations into account and models space utilization as a time-ordered series of points to improve accuracy and biological relevance while adjusting for missing values. BRB estimates the probability of an individual being in a specific location during the study time period and can be used to estimate home range (the area where individuals spend a defined percentage of their time).

To parameterize BRB models for each individual, we considered points collected more than three hours apart to be uncorrelated. However, the two temporally-consecutive points that are deemed uncorrelated by the prior cutoff, may in fact be correlated (e.g., when individuals go to sleep for more than three hours in a single location). Without manually adding points between them, this method will underestimate the usage of homes. An individual is considered stationary when the distance between two consecutive points is less than 10 meters. The minimum standard deviation in relocation uncertainty is set at 30 meters. For each individual, estimation for the usage of different places was done for the whole study period (i.e. for the duration of his/her contribution) and for each season as described below.

In Central and Southern Myanmar, the monsoon rain starts in mid-May and ends in mid-October ^39, 40^. Therefore, we split the data on 15 ^th^ May 2017 and 15 ^th^ October 2017, and the period between the two dates was regarded as the “rainy season”. Mid-October to mid-March is the “cool and dry season”, mid-March to mid-May is the “hot and dry season”. Combinations of the two dry seasons had been used simply as the “dry season” in some of the analyses.

### Ethics statement

Approval for this research project was obtained from the Faculty of Tropical Medicine Ethics Committee, Mahidol University (TMEC 17-007); by the Oxford Tropical Research Ethics Committee (OxTREC reference: 503–17); and by the Tak Province Community Ethics Advisory Board (T-CAB reference: TCAB-04/REV/2016). All participants provided written informed consent in the Karen language.

## Results

A total of 50 persons participated for at least two seasons during the one-year study period. The age and gender distribution of the participants can be found in Table 1. Female participation was low (n= 10). Efforts were made to increase female recruitment but many women declined, stating that they did not normally leave their homes or villages and therefore thought they would not be interesting for the study. Most participants (29 out of 50) were in the 20–40 age group. Individual duration of participation differs between participants ( *Extended data*: Figure S3 ^27^). The mean percentage of days GPS points were actually observed for each participant over the study is 86.17% (median: 91.21, IQR: 79.54 to 96.93).

**Table 1.** Age and gender distribution of the participants.

Age group	Less than 20	20–40	40 and above	Total
**Male**	7	24	9	40
**Female**	2	5	3	10
**Total**	9	29	12	50

During the study period, a 39-year old male was diagnosed with *Plasmodium vivax* malaria. A 45-year old male sustained a non-fatal gunshot injury while going to his farm one early morning. A 30-year old male passed away from the “cancer inside stomach”, the illness that was made known to the field supervisor seven months after being in the study when his mobility became restricted.

### Daily movement ranges

**Figure 1.**
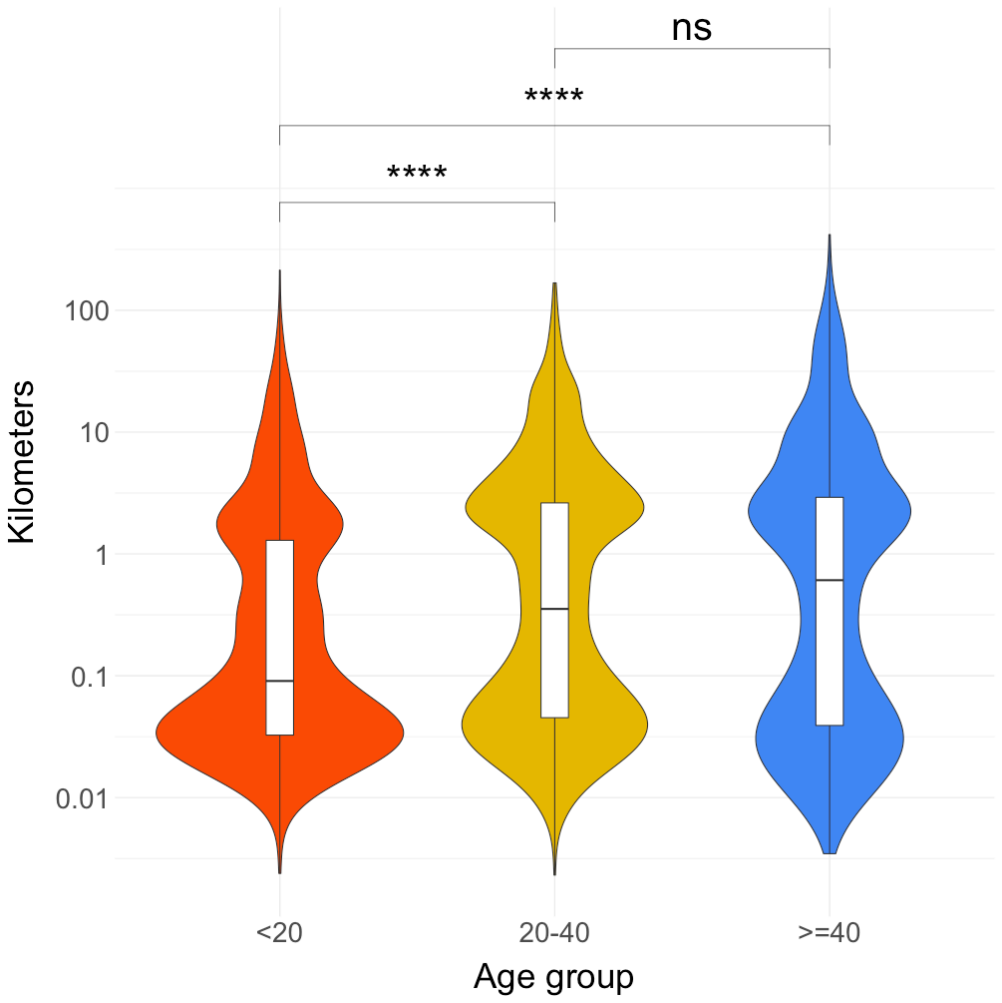
Maximum daily Euclidian distances traveled by participants in kilometers. Distance was calculated from the location a person was at the end of the prior night (most often, this location is their home location). Wilcoxon rank-sum test results are shown on the top of the lines connecting the age groups chosen for the tests. “ns” represents a p-value of > 0.05. **** represents a p-value of <= 0.0001.

The violin plot of the maximum daily Euclidian distances traveled in kilometers in log _10_ scale ( Figure 1) shows that there is a bimodal distribution for all three age groups. The violin plot is a hybrid of kernel density plot and box-plot with the axes flipped that is particularly used to describe data with multimodal distribution. In the figure the vertical axis is the distance value in kilometers with the smallest value at the bottom, and the horizontal axis shows the density value. The heights and peaks in the following results refer to the width/broadness of the violins in the horizontal axis. The first peak was between 0.01 to 0.1 kilometers (10 and 100 meters) and the second peak was between 1 and 10 kilometers. The relative heights of the two peaks differ in different age groups. For under 20s, the first peak is over 20% higher (i.e. they have higher proportion of daily maximum distance close to where they were the previous night) compared to the second peak. The difference between the two peaks in the other two age groups is less than 10%.

The Wilcoxon rank-sum tests provided evidence that 20–40 and over-40 age groups have greater maximum daily Euclidian distances away from home compared to under-20 age group on average. Further disaggregation of this data by gender, and age group can be found in the *Extended data*: Figure S4 ^27^.

### Multiday trips

Participants may make trips that would last several days, either because their destination could not be reached within a single day or because they stayed at their destination for several days (e.g. staying at a farm hut). Using a buffer radius of 266 meters around their home GPS points as their home locations, we calculated the number of consecutive days they spent away from home. Aside from two participants (an over-40 male and an under-20 female), all other participants had at least one trip with more than two consecutive days away from home during their participation period. Trips of less than 10 consecutive days are the most frequent among the participants. There are male outliers of over 20-years old (n=6) who took shorter consecutive day trips (2–5 days) over 10 times. Making trips of over 10 consecutive days was relatively uncommon, but 21 participants still made at least one trip of over 20 consecutive days away from home. Details are available in the *Extended data*: Figure S5 ^27^.

### Days spent at the forest or farm

For each participant, we identified the number of days spent at farms, forests, or at one’s home, and looked for an association between farm visits and forest visits. Here we assumed that having at least two GPS points in the polygon of a particular place constitutes using the respective place for that day, and that a person can be at various types of places in a single day. We found that if a person spent a higher proportion of days at the farms, she or he will likely spend a lower proportion of days at the forests, and vice versa, even though both being at the farms and being in the forests are possible on the same day.

**Figure 2.**
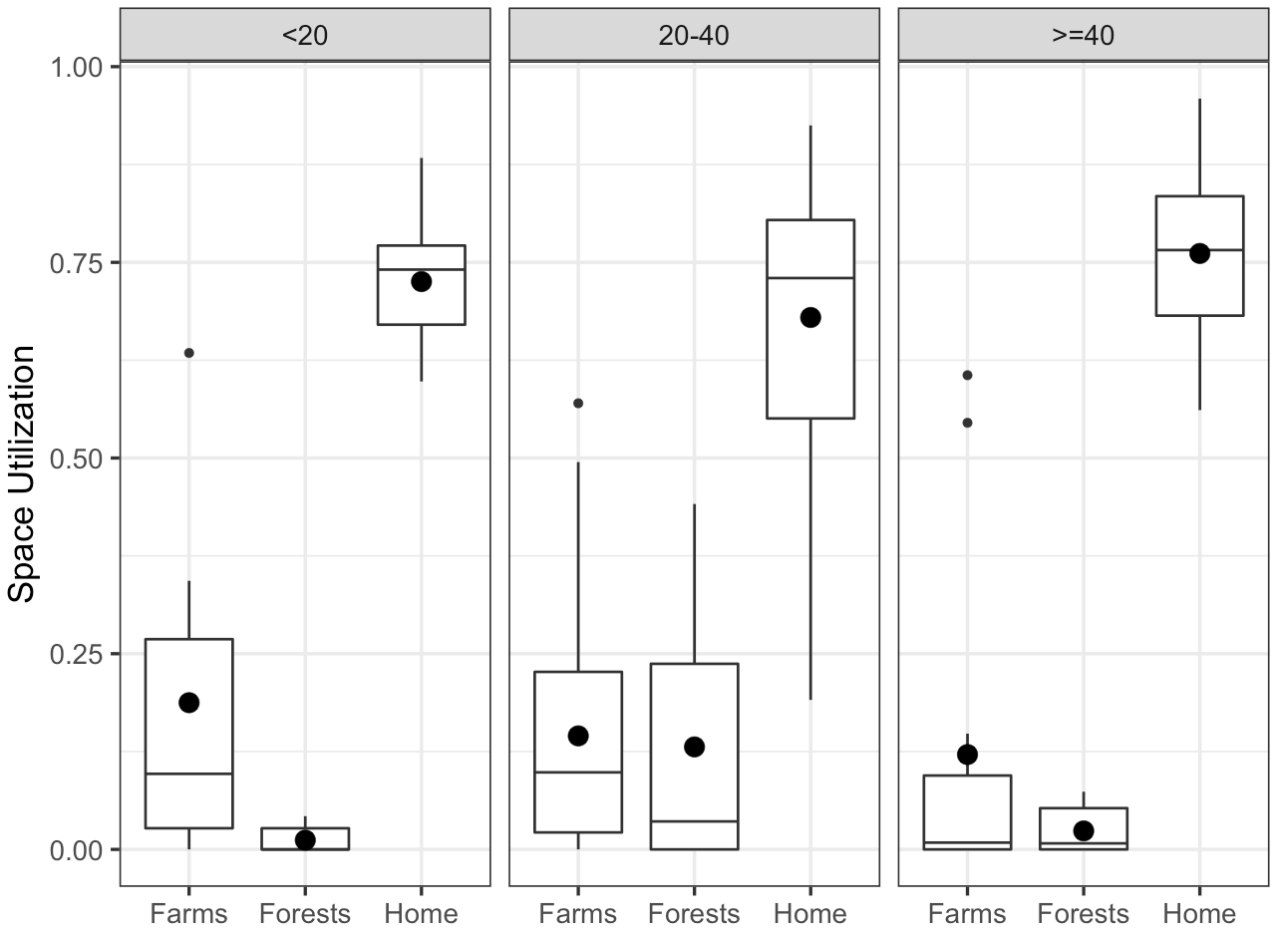
Utilization of the farm, forest, and home (calculated as a proportion of number of days being at the respective places) over the participation period for different age groups. The bigger dots represent the mean values, while the smaller dots represent the outliers.

Figure 2 shows the distribution of the proportion of the number of days being at the farms, forests or home for different age groups. All participants were found to be at their respective home for the majority of days. Compared to other age groups, the 20–40 age group had a higher proportion of time spent in the forests. The under-20 group had the highest proportion of time spent in the farms on average, followed by the 20–40 age group.

### Time spent at the forest or farm

**Figure 3.**
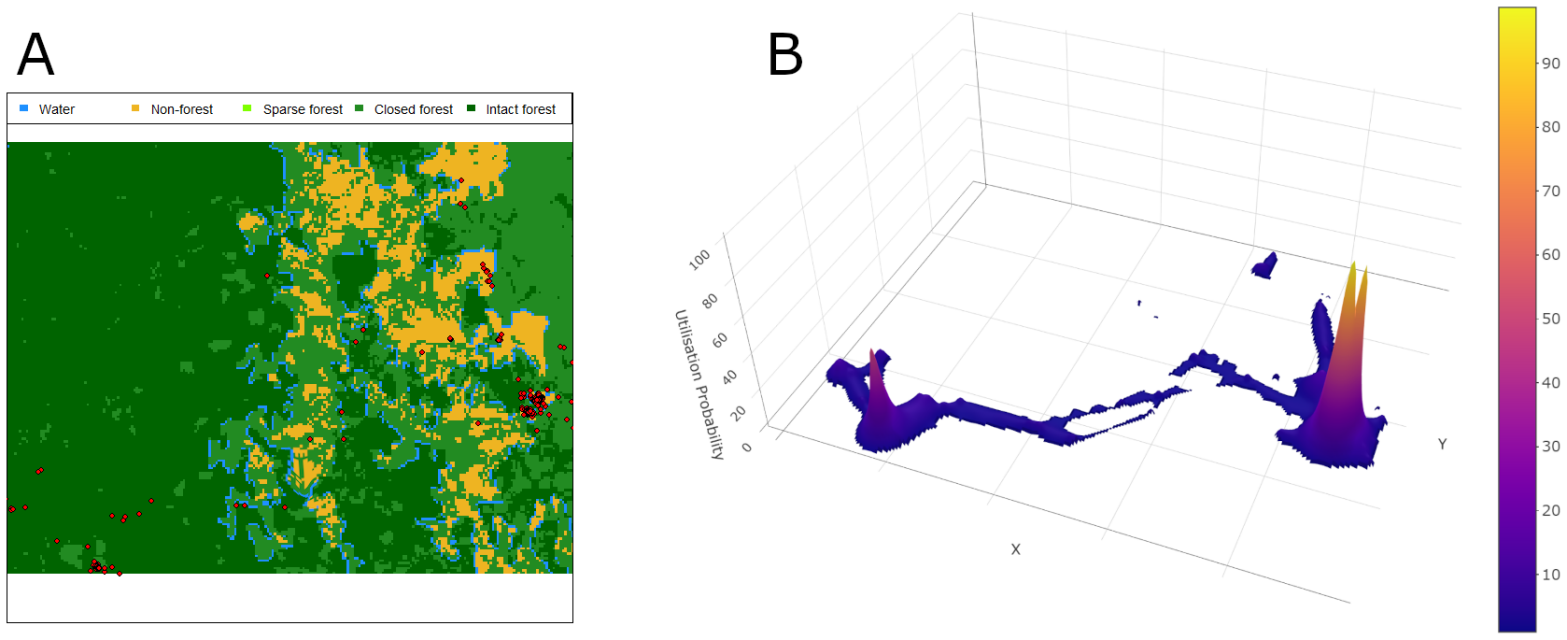
**A**. Example of GPS points (red points) recorded for a person. **B**. Corresponding utilization probability calculated from the GPS points. Its 3D version can be found here.

We also combined the geographic information of farms and forests with the place utilization estimated from a biased-random bridge (BRB) algorithm, and calculated the utilization of each specific place over the study period ( *Extended* data: Figure S6 ^27^). An example of the place utilization of a person can be seen in Figure 3. On average, participants in the under-20 age group spent 20.0% and 2.2% of their time in farms and forests, respectively. For the participants from the 20–40 age group the percentages are 7.6% and 7.4%, and for those in the over-40 age group, the percentages are 7.2% and 3.8%, respectively.

### Nights spent in the forest or farm

**Figure 4.**
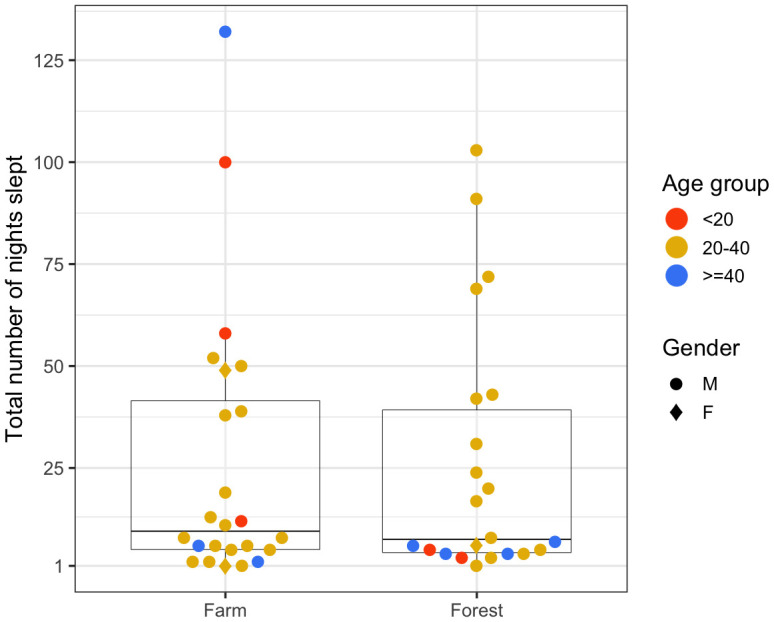
Total number of nights spent in the farms and the forests by each person over the participation period.

Being in the farms and forests at night might impose increased risks of diseases such as malaria because of potential exposure to important mosquito vector species (i.e. *Anopheles dirus)*. As seen in Figure 4, we looked at the total number of nights participants spent in the farms or in the forests. Two female participants (20% of females) spent at least a night in the farm compared to 22 male participants (55% of males). As for spending at least a night in the forest, there were 21 males and only one female. Most participants in the 20–40 age group spent at least one night in the farm (18 out of 29, 62%) and in the forest (16 out of 29, 55%) whereas fewer than 35% of participants from under-20 and over-40 age groups spent a night in such places.

The negative binomial regression provided strong evidence that males in this cohort were more likely to spend nights in farms (p=0.045) and in forests (p=0.01) compared to females, and that young adults (the 20–40 age group) were more likely to spend nights in the forest compared to the under-20 age group (p=0.043), after controlling for the remaining variables ( Table 2).

**Table 2.** Association between number of nights slept in farms or forests and age, gender, and season.

	*Nights slept in farms*		*Nights slept in forests*	
*Predictors*	*Incidence rate ratios*	*p-value*	*Incidence rate ratios*	*p-value*
*(Intercept)*	0.06 [0.00 – 2.01]	0.116	0.00 [0.00 – 0.17]	**0.005**
*Age [<20]: comparator*				
*Age [>=40]*	0.15 [0.01 – 3.24]	0.228	2.03 [0.09 – 46.25]	0.658
*Age 20–40*	1.43 [0.11 – 18.52]	0.784	16.80 [1.09 – 259.75]	**0.043**
*Gender [F]: comparator*				
*Gender [M]*	14.80 [1.07 – 205.70]	**0.045**	46.34 [2.47 – 869.07]	**0.010**
*Season [Dry]: comparator*				
*Season [Rainy]*	1.20 [0.66 – 2.19]	0.554	0.74 [0.35 – 1.58]	0.435
***Random effects***				
*σ ^2^*	1.35		1.66	
*τ _00_*	6.62 _pid_		5.66 _pid_	
*ICC*	0.83		0.77	
*N*	47 _pid_		47 _pid_	
*Observations*	89		89	
*Marginal R ^2^ / Conditional R ^2^*	0.210 / 0.866		0.341 / 0.850	

**Figure 5.**
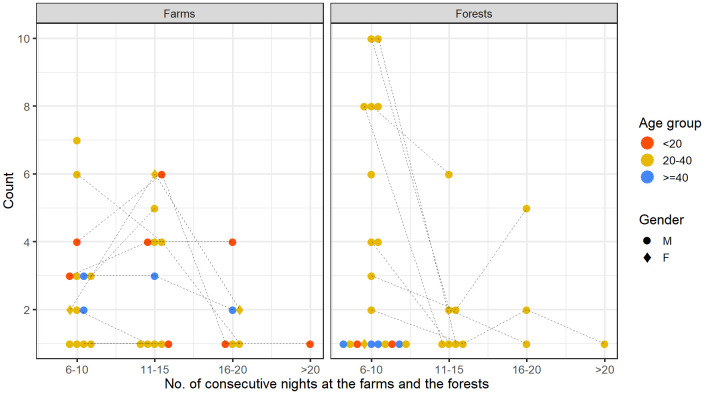
Number of consecutive nights spent in the farms and forests. In each panel, each of the points in each column represents a person of a specific age group and gender, defined in the legend. A single person may contribute a point in each of the columns (e.g., In the panel named Farms, a single person may contribute one point for each of the ranges of consecutive nights). Dotted lines connect the points contributed by the same person across different columns.

Participants may spend consecutive nights in the farms or the forests without going back home. The number of consecutive nights spent in the farms or the forests is the subset of the multiday trips mentioned in the previous section. Figure 5 quantifies this metric for different age groups and gender. Persons of all age groups and gender spent varying numbers of consecutive nights in the farms. An under-20 male spent the most consecutive nights (16–20 nights) in the farm. A female of 20–40 age-group and a male of over-40 age-group spent two episodes of 11–15 consecutive nights in the farm. In contrast, there was little demographic heterogeneity among those who spent consecutive nights in the forests. A few males of the 20–40 age group not only spent long periods of consecutive nights (more than six consecutive nights), but also frequently spent many short periods of consecutive nights (two to five nights) in the forests.

## Discussion

Many detailed human movement studies have been done, mainly in the regions of high socio-economic status. Our study presents an analysis of human movement in a remote rural area that has been under-studied with regard to human ecology (though do see 41, 42). Compared to other studies where GPS loggers were used for a very short period of time, there is a relatively long duration of participation in our study. This makes it possible to examine potential seasonal variation.

Our data suggest a bimodal pattern of movement away from participant homes, with one peak nearby (≤ 100m) and another one to three kilometers away from their homes ( Figure 1). There were differences in these movement patterns by demography, with under-20s staying close to home on the majority of the days and both 20–40 and over-40 age groups tending to move farther away each day. We hypothesize that the reason for this difference is that over-20 age groups are more heavily involved in subsistence activities (e.g., farming and foraging which are conducted further away from home) than the under-20 age group.

Multiday trips of less than 10 days are common among the participants. The metrics of multiday trips do not signify anything unless they are associated with the activities done during the trip which vary from visits to friends/family, getting supplies at the nearby town, farming, foraging, and other economic or subsistence activities.

All age groups in this study visited farm areas and spent the night in the farms, with no statistically significant difference found between age groups. When they spent their nights in the farms, they did it consecutively and on several occasions during the study period. Farming is one of the major forms of subsistence for rural families and it must be regarded as relatively safe compared to subsistence activities in the forests that all age groups partake in it. There was no seasonal variation in the number of nights spent at the farms in these data. Different types of crops are normally rotated over the year for cultivation in this region. 

In contrast, going to and sleeping in the forests, which may involve foraging, logging, mining etc., is found to be the task for males of the 20–40 age group. The median number of nights slept in the forest among those who ever spent the night in the forest was 7.5. Only males of the 20–40 age group spent a higher number of nights in the forest than the median value. The same males (20–40 age group) were found to take frequent and successive overnight trips to the forests. We surmise that the males in the 20–40 age group, most likely being the breadwinners of the family, are subject to any possible subsistence activities and are regarded as the most suitable persons to venture into the forests overnight despite dangers from wildlife and harsh living conditions. No seasonal variation was found in the number of nights of sleeping in the forest. In comparison, a questionnaire based movement survey conducted in similar Thai-Myanmar border area found seasonal movement patterns ^15^.

Compared to home, sleeping places in the farms and forests may be more rudimentary, leaving people more vulnerable to medically important arthropods or other environmental risks (i.e. potentially more contact with venomous snakes, etc.). Spending several consecutive nights in the farms and forests may increase the chances of vector-borne diseases such as malaria since major malaria vectors in the area such as *Anopheles dirus*, and *Anopheles minimus* are found in the deep forests, forest edges, plantations and even in the rice fields ^43^. Studies have found that the increased risk of malaria in forest-goers is contributed by inconsistent bed net usage, misconception that alcohol consumption or blankets provides protection against mosquito bites, non-participation in the malaria prevention activities held at the villages ^44^. 

Results from this study, particularly the space utilization data, would be useful in spatially explicit individual-based infectious disease model such as ^45^ which models the malaria elimination in the rural South East Asian region. Human mobility is a crucial part of many disease transmission dynamics, yet it has been ignored in many infectious disease models because of constraints on data and computational capacity. Compartmental models assume homogeneous mixing of individuals in their respective compartments. While they are quick to set up, they are not suitable for the disease elimination settings. Their homogeneous nature limits the modelers from exploring the impact of multiple interventions tailored towards different risk groups such as forest-goers in malaria intervention. Individual-based models could have individual specific properties and their related movement patterns thus achieving a heterogeneous population. Calibrating on the space utilization data of this study, such models could become more realistic in terms of transmission dynamics. They could provide more accurate and precise estimations to tackle infectious diseases cost-effectively.

The study has several limitations. It was a pilot study and had a limited sample size. Most participants were adult males. Potential female participants said they rarely go beyond village boundaries and thus were not eligible to be included in the study. It may have introduced a selection bias, but it points to the fact that the mobility preferences between the two genders were too different that they were essentially two different populations requiring separate analyses. The most commonly reported occupation was farming and most people in this study area, indeed, farm for at least part of the year. However, people in the study area usually perform different types of work according to the season and assigning a single occupation to a person may not be appropriate. Employment in this region is almost entirely informal, and most working-age men will work in agriculture for part of the year and in other types of labor during other parts of the year. Responses to surveys about employment will therefore vary by the time of year, even within a single research participant.

While we believe that this cohort is representative of adult males in this setting, more studies that are demographically representative of rural villages in this setting could be useful for understanding differences in travel patterns by age and gender. Mobile GPS devices have their own limitations. As explored in the Extended data: Figure S2, their readings could be inaccurate. Because of their small size, their battery capacity was limited. During the study period, participants may have failed to carry the GPS device (intentionally if they engaged in activities that other people might think were illegal or sensitive in nature – or not). Mechanical failures may also cause problems in data collection. Even though the utmost care was taken to preserve data integrity, there could be errors and bias from data collection (due to device inaccuracies) or data manipulation. (described in the methods section under analysis and in *Extended data* Figure 2 ^27^). Categorization of land types such as farms and forests was done manually using satellite imagery. While the categories do match our authors’ understanding of the area, no validation was done on the ground after categorization for this analysis. Our estimation of home location as the median center of all the GPS points where the participant spent the night, each of which in turn is derived from the last GPS point of the day between 6pm to 12 midnight, may not be robust enough to capture the actual home location. This could be overcome by having the field supervisors record each participant’s home location with a GPS device in the future studies. Categorization of home area (266 meters around home location) may be too wide to discern land use that is very close to home.

Finally, the estimation of land utilization regardless of the method used is imperfect. Having two consecutive GPS points to constitute usage of the land area provide too crude a result ( Figure 2). While the BRB method provide more accurate and precise estimates (Extended data: Figure S6), it is not without its caveats. The BRB approach assumes that consecutive points that were more than three hours apart were uncorrelated. Since the GPS logger went into sleep mode while stationary, the current land utilization estimation under-estimates the time spent motionless (e.g., sleeping) and hence resulting in lower usage of home in Extended data: Figure S6 compared to that in Figure 2.

## Conclusion

This study shows that it is feasible to use GPS loggers to document and quantify human movement patterns in this setting (the Thailand-Myanmar border). Most individuals who agreed to participate did so across multiple seasons. Further work using GPS loggers in this setting is likely feasible. We found that younger age groups spent more days around their home compared to older age groups. Older age groups spent almost equal amounts of time both around their home and at places one to three kilometers away from their home. Males spent more nights in the farms and forests, especially those in the 20–40 age group. The resulting human movement characteristics can be incorporated in infectious disease modeling studies in similar regions and the operational and analytic lessons learned from this project are broadly applicable to other studies of human movement and travel.

## Data Availability

Demographic data on participants and participant drop out are provided in the manuscript. All R code used in this analysis is available at: https://github.com/SaiTheinThanTun/HumMovPatt. The time-stamped locational data are restricted for confidentiality reasons (i.e. it would be possible to identify the location of participant homes with these data). Access to sanitized data, through which participant home locations cannot be identified, will be considered on a case-by-case basis. Please contact Daniel M. Parker ( dparker1@hs.uci.edu) with queries about data access All R code for this analysis is available at: https://github.com/SaiTheinThanTun/HumMovPatt This project contains the following extended data: Figure S1 (Study site and location of clinics that were used for recruitment) 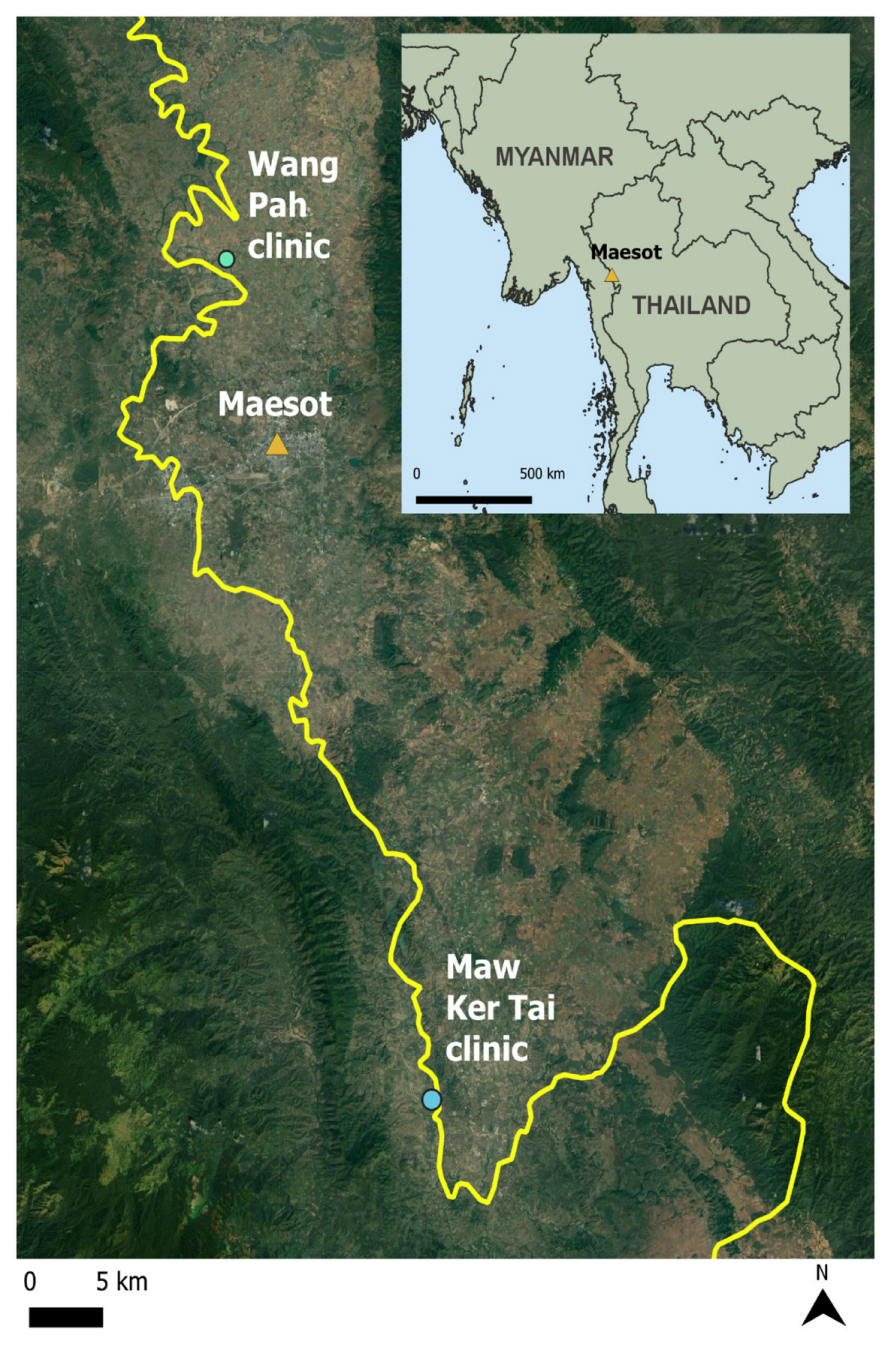 Figure S2 (GPS reading errors in stationary devices) 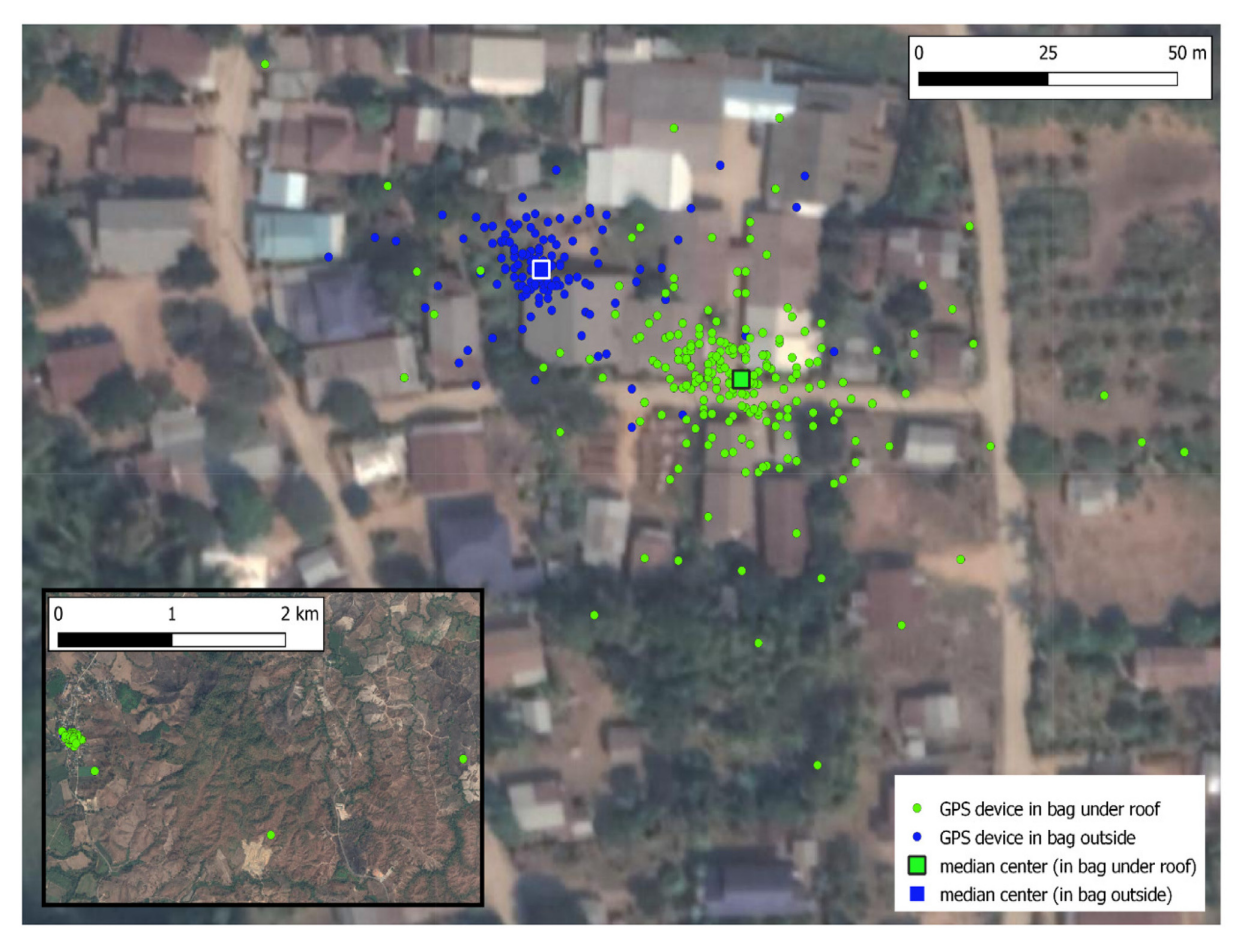 We conducted field tests of GPS device error under stationary conditions (Figure S2). These tests consisted of placing GPS loggers in stationary locations (tied to a bamboo pole, on a shelf in a house), plotting the points from the device over a period of one week, and measuring the geographic distribution of those points from their geographic centre. Devices were placed inside bags, as this would also be likely for carriage/storage by participants. The mean locational error recordings was larger for the in-doors device. Most erroneous points were within 50 meters of the house. However, a few points were far outside of this range (inset map on bottom left). The maximum distance away from the centre for any of the erroneous points was over 3km away. Only one reading was recorded at this distance and the next reading (30 minutes later) was back within the 50m radius around the house. We calculated a standard deviation from the median centre for the worst performing test (in a bag, inside a house) of 266m radius and used this as a basis for judging whether or not a participant’s movements were likely real or the result of measurement error. This is a conservative estimate. Figure S3 (Duration of participation for each person, over the study period) 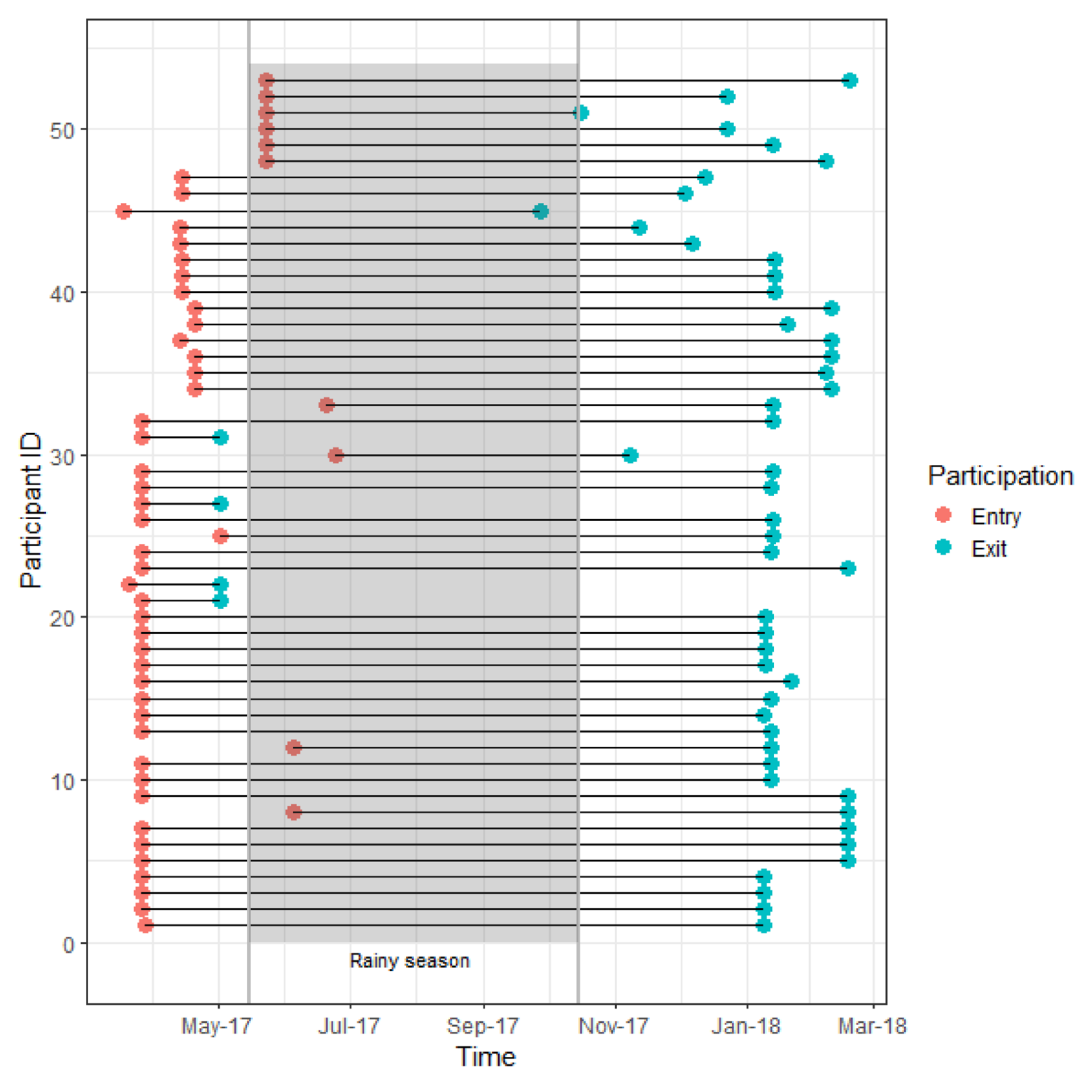 Figure S4 (Frequency histogram of maximum Euclidian distance travelled by the participants in kilometres) 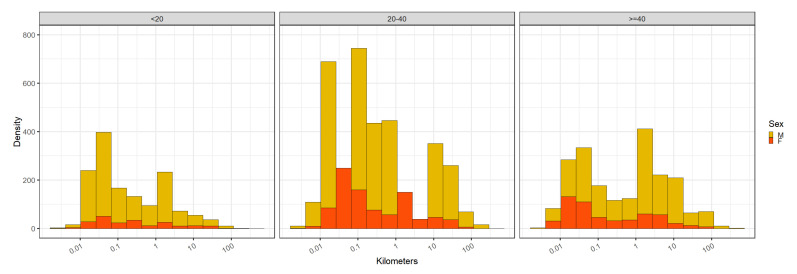 Figure S5 (Multiday trips made by the participants) 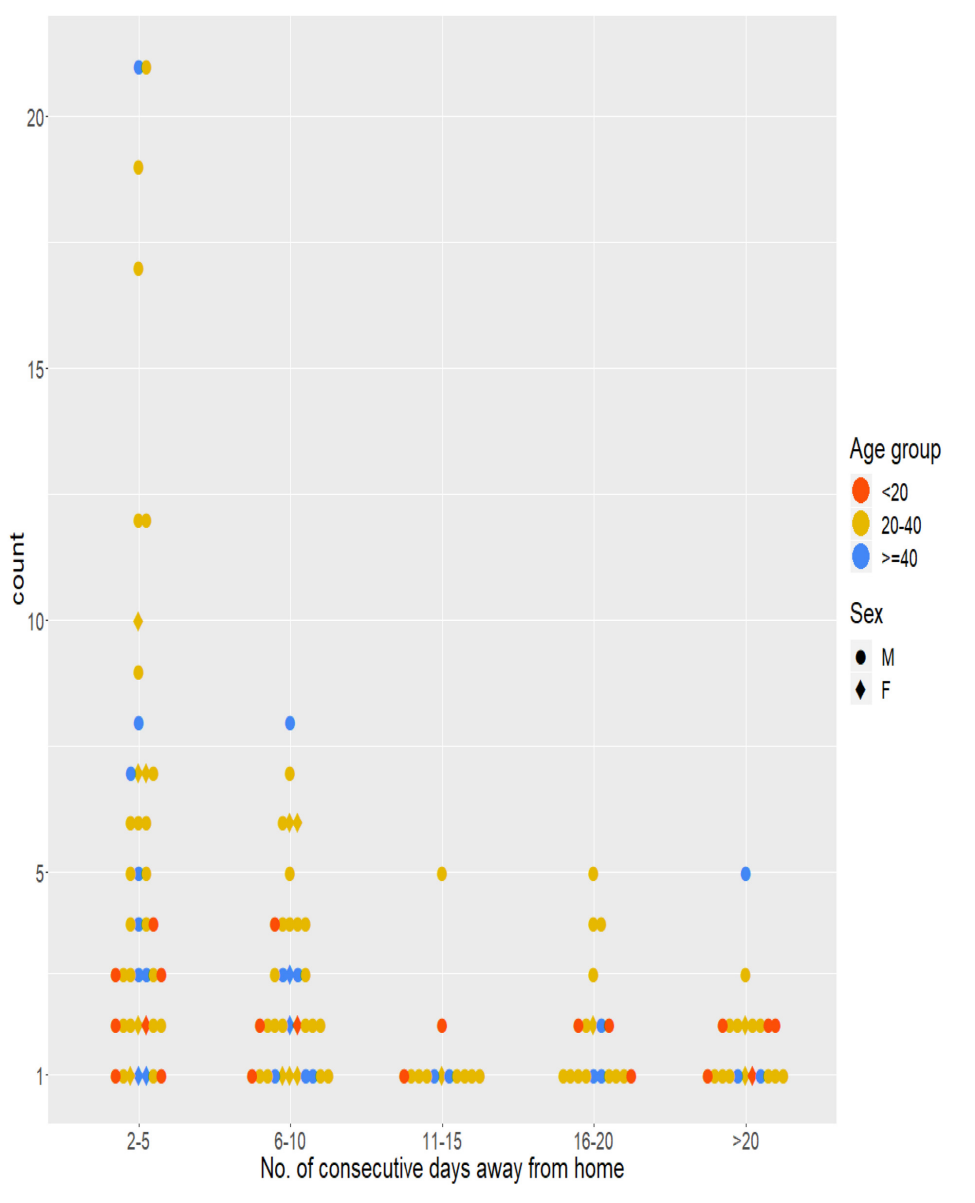 Figure S6 (Utilization of the farm, forest, and home over the participation period for different age groups) 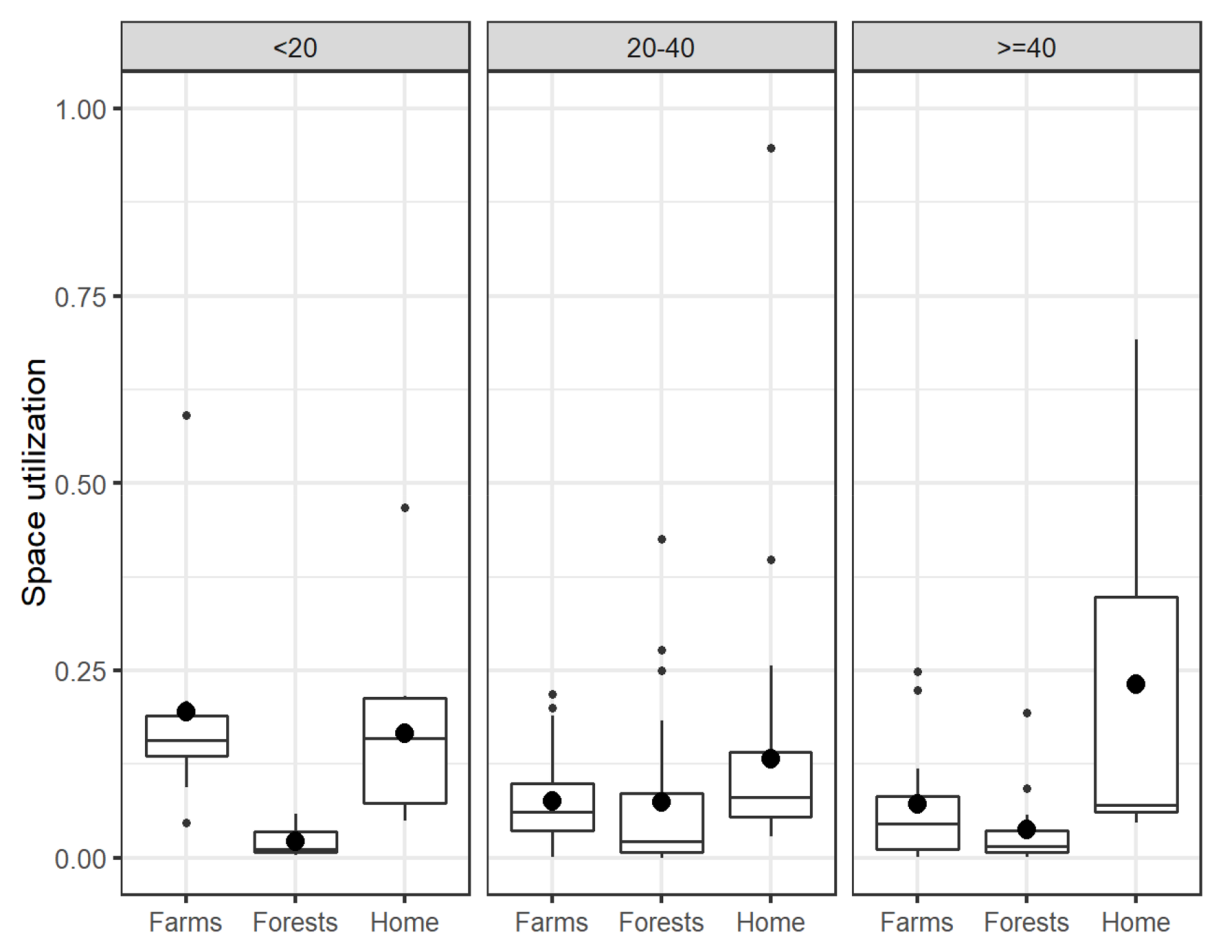 The bigger dots represent the mean values, while the smaller dots represent the outliers. Usage of Home was underestimated because of the limitation explained in the Methods section. Data are available under the terms of the Creative Commons Attribution 4.0 International license (CC-BY 4.0).

## Code availability

Analysis code available from: https://github.com/SaiTheinThanTun/HumMovPatt/tree/v1.0.1

Archived analysis code at time of publication: https://doi.org/10.5281/zenodo.4782737 ^27^.

License: MIT
