# Supplementary material for: Human movement patterns of farmers and forest workers from the Thailand-Myanmar border
Source: Wellcome Open Res. 2023 Oct 2;6:148. Originally published 2021 Jun 14. [Version 2] doi: 10.12688/wellcomeopenres.16784.2 (PMC10660292; doi:10.12688/wellcomeopenres.16784.2)
Supplement: Peer review history

# Reviewer report: Reviewer response for version 2

**DOI:** 10.21956/wellcomeopenres.22276.r68377

**Published:** 2023-11-20

**Authors:** Yao Li

**Affiliations:** 1 Department of Geographical Sciences, University of Maryland at College Park, College Park, Maryland, USA; 2 Department of Epidemiology, Johns Hopkins Bloomberg School of Public Health, Baltimore, Maryland, USA

Q1. Citations are not current,

 There are multiple studies studying the human movement in Thailand and Myanmar under the context of malaria very recently, and it is better for authors to cite these papers.

 Q2. “These clinics primarily serve migrant and cross-border populations and have connections to village health workers in nearby villages. We reached out to village health workers in the nearby villages to explain the project and to ask if they could help us recruit participants from their respective villages.”

 Are you recruiting local people, or do you include all the people who are willing to participate, potentially including migrants from Myanmar?

 Q3. The conclusion is different from the abstract

 In the abstract, the author mentioned the “results from this study are useful for individual-based models of disease transmission and land use.” However, in the conclusion paragraph, I didn’t see any description of land use.

Is the work clearly and accurately presented and does it cite the current literature?

Partly

If applicable, is the statistical analysis and its interpretation appropriate?

Yes

Are all the source data underlying the results available to ensure full reproducibility?

Yes

Is the study design appropriate and is the work technically sound?

Yes

Are the conclusions drawn adequately supported by the results?

Partly

Are sufficient details of methods and analysis provided to allow replication by others?

Yes

Reviewer Expertise:

Infectious diseases, Geographical Sciences, Malaria, human mobility.

I confirm that I have read this submission and believe that I have an appropriate level of expertise to confirm that it is of an acceptable scientific standard, however I have significant reservations, as outlined above.

# Reviewer report: Reviewer response for version 2

**DOI:** 10.21956/wellcomeopenres.22276.r68388

**Published:** 2023-11-20

**Authors:** Nick Ruktanonchai

**Affiliations:** 1 Department of Population Health Sciences, Virginia Tech, Blacksburg, VA, USA

I believe the issues brought up by previous reviewers have been sufficiently addressed. I might be inclined to re-introduce the importance of studying the target population--this region is important for malaria elimination as noted by the authors, but it also highlights the importance of the groups that *weren't* effectively reached by the study authors, notably women. Certainly I think it is worth noting that there is a bias among participants of research on human mobility that researchers want to understand the travel patterns of only people who travel a lot, and this introduces biases to our understanding of human mobility in many communities across the Global South.

Is the work clearly and accurately presented and does it cite the current literature?

Yes

If applicable, is the statistical analysis and its interpretation appropriate?

Yes

Are all the source data underlying the results available to ensure full reproducibility?

Yes

Is the study design appropriate and is the work technically sound?

Yes

Are the conclusions drawn adequately supported by the results?

Yes

Are sufficient details of methods and analysis provided to allow replication by others?

Yes

Reviewer Expertise:

NA

I confirm that I have read this submission and believe that I have an appropriate level of expertise to confirm that it is of an acceptable scientific standard.

# Reviewer report: Reviewer response for version 2

**DOI:** 10.21956/wellcomeopenres.22276.r67940

**Published:** 2023-10-23

**Authors:** Lisa Sattenspiel

**Affiliations:** 1 Department of Anthropology, University of Missouri, Columbia, MI, USA

My previous comments have been addressed in this version. I realize this paper presents the results of a preliminary feasibility study of mobility patterns and I think the research indicates that it is possible to use the methods proposed to collect valuable information on daily mobility patterns in a rural and less developed region. I would have liked to see a little more in-depth discussion of the insights related to the impact of the observed mobility patterns on disease transmission patterns that have been gained from this study and/or would be gained from a more extensive study using the proposed methods.

Is the work clearly and accurately presented and does it cite the current literature?

Yes

If applicable, is the statistical analysis and its interpretation appropriate?

Partly

Are all the source data underlying the results available to ensure full reproducibility?

Partly

Is the study design appropriate and is the work technically sound?

Yes

Are the conclusions drawn adequately supported by the results?

Partly

Are sufficient details of methods and analysis provided to allow replication by others?

Partly

Reviewer Expertise:

human infectious diseases, agent-based modeling, anthropology, mobility and infectious disease

I confirm that I have read this submission and believe that I have an appropriate level of expertise to confirm that it is of an acceptable scientific standard.

# Reviewer report: Reviewer response for version 1

**DOI:** 10.21956/wellcomeopenres.18514.r53656

**Published:** 2023-01-13

**Authors:** Lisa Sattenspiel

**Affiliations:** 1 Department of Anthropology, University of Missouri, Columbia, MI, USA

This paper presents the results of a pilot study to test the ability of wearable GPS loggers to track the movements of rural farmers in a region along the border of Thailand and Myanmar. The underlying purpose is to assess whether this type of equipment can adequately provide detailed data on human activities that can be used to better inform studies of infectious disease transmission and spread. A second purpose is to assess the feasibility of the technology to collect the detailed data that would be needed to adequately parameterize individual-level models of human activities and disease transmission. The main goal of the paper is primarily to describe the nature of human movement that was measured using the GPS technology, however, and it was not focused on either disease transmission or disease models.

 I found the study very interesting and the results encouraging. I agree with the authors that there is a bias in knowledge about human mobility toward data derived from studies of urban populations, and the activities engaged in by the many small-scale societies living under traditional subsistence strategies such as small-scale farming or foraging are much less understood. These types of societies are common in many parts of the world however, and they are especially common in tropical regions with high risks for infectious disease transmission and very low levels of development and resources. Understanding more about the specific activities that increase potential for disease transmission may help not only the residents of the small-scale societies, but also people in many other regions, because these environments have significant potential for the evolution of new and emerging pathogens.

 I have several comments and concerns that I think should be addressed in the manuscript, however. (Page numbers refer to the pdf version of the article)

1. The context of the study needs to be described at the very outset so that the reader can visualize the setting in which the measurements are being taken. What is the geography of the area (e.g., hilly, mountainous, lowland, etc.), what are the houses like, what distinguishes a farm from forest, what activities are performed in these different areas, etc.? Since the ultimate goal is to better understand how the movement patterns may impact infectious disease transmission, it is essential to have some understanding of the setting within which both the movement and potential disease transmission occurs.
2. The authors mention other methods that have been used to collect data on human movement, including surveys and mobile phone records. Important limitations of these methods are mentioned, but there is no discussion of the limitations of the wearable GPS technology. In particular, the authors mention that some types of movement may not be captured by survey methods, especially those of a sensitive nature. This concern holds just as much for wearable GPS devices – people engaging in illegal or sensitive activities may either not wear the devices on certain days or refuse to engage in the study altogether. The article would benefit by a more substantive discussion of both the pros and cons of each of the methods, not just the cons of other methods and the pros of the wearable technology.
3. Have any procedures been implemented to ensure that the GPS loggers are always being worn by the intended persons?
4. The authors mention that they gathered information on illnesses during their monthly meetings. Although the goal of this paper is to discuss the data that were collected on movement patterns, since these data are being collected to eventually help assess disease risks and patterns, it would be useful to have a brief discussion of the kind of illness information that was collected during the pilot study and the underlying reasons for the collection of that information.
5. The description of the two methods used to estimate the utilization of places was a little unclear, especially for someone like myself who is not familiar with the methods. Some clarification would be helpful.
6. The authors mention the strong sex bias in the participants. The potential implications of this bias should be addressed fully in the discussion section. Was it at all related to whether a request for participation was made by a male vs. female researcher? How does the lack of female participation influence the results and the prospects for a full-scale study? If such a bias cannot be sufficiently overcome, what are the implications of this for the data that have been and will be collected?
7. The authors should provide a brief explanation of violin plots and how they should be examined. For example, it would help to explain that the "heights" of the peaks are the amount they extend out to the left or right, not the vertical distance. In addition, the values mentioned in the results on daily movement ranges (i.e., the first peak is between 10 and 100 km and the second between 1 and 10 km – p. 5) seem inconsistent with what is shown in Figure 1. To me it looks like the first was between 1 and 10 km and the second between 0.01 and 0.1 (although the authors actually refer to the first peak being the larger one for the <20, so that should be the one between 0.01 and 0.1 and the second peak should be the one between 1 and 10 km). These results are mentioned again in the discussion section on p. 9, but in that case they do seem consistent with what is observed in Figure 1.
8. Was any information gathered about the reasons for multiday trips? Besides being longer (and presumably further) were the activities engaged in similar or different to the single day trips? What implications do multiday trips have for understanding the potential for infectious disease transmission?
9. Some additional discussion of the reasons for the movement patterns that were observed should be added to the discussion section of the paper. For example, in the results section it is noted that spending more time at the farms usually meant less time in the forests (p. 5) and it is also noted that 20-40 year olds spend the most time in the forest (p. 6). Why is this? How does it relate to the potential for disease transmission?
10. Explain why the pictures in Figure 2 and Figure S6 differ from each other, since both are measuring the utilization of different places.
11. The discussions of the implications of the observed movement patterns for transmission of diseases prevalent in the study environment needs to be fleshed out. Although this paper is designed to present the results derived from the GPS loggers, there should be a full discussion of what the preliminary data from this study might suggest about how the mobility in the region directly affects disease transmission. Assuming the authors can use their results to derive hypotheses to test in future studies, the discussion is a good place to present those ideas.
12. The paper mentions the importance of the data for “calibrat[ing] human movement patterns in models of infectious diseases”, but there is no discussion at all of what such models are like, what kinds of data are needed in such models, and how the data from the GPS loggers can help calibrate those models. There should either be a substantive discussion of this issue or the use of the data for modeling disease spread should be omitted from the paper at this time.
13. Finally, on p. 9 the authors mention some limitations related to occupation. However nowhere previous to this in the paper has there been any discussion of occupation. A discussion of the normal activities/occupations of the participants should be included in the introductory material suggested in #1 above. Then, the discussion of occupation as a limitation of the pilot study should be elaborated so it is clear why this is an important issue.

Is the work clearly and accurately presented and does it cite the current literature?

Yes

If applicable, is the statistical analysis and its interpretation appropriate?

Partly

Are all the source data underlying the results available to ensure full reproducibility?

Partly

Is the study design appropriate and is the work technically sound?

Yes

Are the conclusions drawn adequately supported by the results?

Partly

Are sufficient details of methods and analysis provided to allow replication by others?

Partly

Reviewer Expertise:

Human infectious diseases, agent-based modeling, anthropology, the impact of daily mobility on infectious disease transmission

I confirm that I have read this submission and believe that I have an appropriate level of expertise to confirm that it is of an acceptable scientific standard, however I have significant reservations, as outlined above.

# Response

**Published:** 2023-10-23

**Authors:** Daniel Parker

**Affiliations:** University of California, Irvine, USA

Dear Editors and Reviewers; Thank you for your helpful comments and suggestions. We would also like to take this opportunity to apologize for our slow movement on addressing your comments and critiques. In the last few years there has been a military coup in Myanmar, on top of the extreme challenges that the pandemic of SARS-CoV-2 has presented, and this has delayed us in our focus on this manuscript and in many other ways. We have also been experiencing some debilitating health issues that have slowed our work. We very much appreciate the   time and effort you put into reviewing our manuscript and we hope that we’ve made it clear that our delays in addressing these comments are because of external factors.

 We have attempted to address each of your comments and suggestions and we believe that the manuscript is now much improved. Please see our line-by-line responses below: 1.    The context of the study needs to be described at the very outset so that the reader can visualize the setting in which the measurements are being taken. What is the geography of the area (e.g., hilly, mountainous, lowland, etc.), what are the houses like, what distinguishes a farm from forest, what activities are performed in these different areas, etc.? Since the ultimate goal is to better understand how the movement patterns may impact infectious disease transmission, it is essential to have some understanding of the setting within which both the movement and potential disease transmission occurs.

 We have added a new section named “Context of the study area”:

 “The study area is on the Thailand-Myanmar border. Participants were recruited from clinics that serve rural, mostly underdeveloped, and low population density communities. Most participants were of the Karen ethnic group. Villages were made up of a few dozen of mostly multigeneration families living in stilt houses made of wood and thatched roof. Villages didn’t normally have schools, clinics, or sanatory toilets. The houses are normally located along the main dirt road of the village. The dirt roads then continued to connect to other villages and small towns through a hilly and rugged terrain with occasional watersheds and river basins which made traversing difficult, especially during the rainy season.  2.    Villagers made their living mostly though agriculture, but they have to undertake various types of jobs throughout the year for their subsistence. They developed land in and around their villages into farms to cultivate rice and vegetables. They farm poultry and pigs under their stilt houses. Some villagers go into the forests for hunting, or for foraging wood, and to collect wild edibles. They would go to the farms and forests overnight occasionally, and sleep without much protection from mosquitos and insects. We focused on farms and forests as places of interest in this study since apart from their homes, farms and forests might be the places the villagers spend significant amount of their time while being vulnerable to infectious diseases such as malaria.”The authors mention other methods that have been used to collect data on human movement, including surveys and mobile phone records. Important limitations of these methods are mentioned, but there is no discussion of the limitations of the wearable GPS technology. In particular, the authors mention that some types of movement may not be captured by survey methods, especially those of a sensitive nature. This concern holds just as much for wearable GPS devices – people engaging in illegal or sensitive activities may either not wear the devices on certain days or refuse to engage in the study altogether. The article would benefit by a more substantive discussion of both the pros and cons of each of the methods, not just the cons of other methods and the pros of the wearable technology.

 We agree with the limitations of the wearable GPS technology you have pointed out. They have been added to the Discussion section in appropriate places. 3.    Have any procedures been implemented to ensure that the GPS loggers are always being worn by the intended persons? When the field managers met with the participants on a monthly basis during the study, their general movement patterns were asked and checked against the information from the GPS loggers. Apart from this monthly validation, we cannot enforce the loggers to be worn only by the intended persons.

 4.    The authors mention that they gathered information on illnesses during their monthly meetings. Although the goal of this paper is to discuss the data that were collected on movement patterns, since these data are being collected to eventually help assess disease risks and patterns, it would be useful to have a brief discussion of the kind of illness information that was collected during the pilot study and the underlying reasons for the collection of that information.

   

 During the study period, a 39-year old male was diagnosed with Plasmodium vivax malaria. A 45-year old male sustained a non-fatal gunshot injury while going to his farm one early morning. A 30-year old male passed away from the “cancer inside stomach”, the illness that was made known to the field supervisor seven months after being in the study when his mobility became restricted. 5.    The description of the two methods used to estimate the utilization of places was a little unclear, especially for someone like myself who is not familiar with the methods. Some clarification would be helpful. We have revised the description of the two methods for better clarity as follows:

 “Utilization of places (home, farms or forests) for each person was estimated using two different approaches. The first method was by checking whether more than two temporally-consecutive GPS points of a person fall within a polygon (a GIS object with a series of x and y coordinate pairs that represents an enclosed area on a map) designated for the person’s home (for this particular measurement, the polygon is a circle of radius 266 meters around the person’s home location), farms, or forests on each day. This is equivalent to checking if a person spent at least an hour within the same polygon. For each participant, the number of days spending in each category of place (home, farm, forest) was divided by the total number of days participated during the study period to obtain the proportion of being at the respective places. The second method estimated the utilization of places by a biased random bridge (BRB) technique. Unlike prior methods for estimation of utilization of places such as location-based kernel density estimations (LKDE), BRB takes the activity time between successive relocations into account and models space utilization as a time-ordered series of points to improve accuracy and biological relevance while adjusting for missing values. BRB estimate the probability of an individual being in a specific location during the study time period and can be used to estimate home range (the area where individuals spend a defined percentage of their time).”

 6.    The authors mention the strong sex bias in the participants. The potential implications of this bias should be addressed fully in the discussion section. Was it at all related to whether a request for participation was made by a male vs. female researcher? How does the lack of female participation influence the results and the prospects for a full-scale study? If such a bias cannot be sufficiently overcome, what are the implications of this for the data that have been and will be collected?

 Request for participation was made by male researchers only and this could have influenced the recruitment. We did attempt to boost female participation, but potential female participants responded that they rarely go beyond village boundaries which is one of the exclusion criteria of the study. As such, we are quite limited in analyzing female movement patterns and in future studies would put more effort into recruiting more women participants (also through recruiting female staff to work on the project). 

 7.    The authors should provide a brief explanation of violin plots and how they should be examined. For example, it would help to explain that the "heights" of the peaks are the amount they extend out to the left or right, not the vertical distance. In addition, the values mentioned in the results on daily movement ranges (i.e., the first peak is between 10 and 100 km and the second between 1 and 10 km – p. 5) seem inconsistent with what is shown in Figure 1. To me it looks like the first was between 1 and 10 km and the second between 0.01 and 0.1 (although the authors actually refer to the first peak being the larger one for the <20, so that should be the one between 0.01 and 0.1 and the second peak should be the one between 1 and 10 km). These results are mentioned again in the discussion section on p. 9, but in that case they do seem consistent with what is observed in Figure 1.

 We have now added an explanation of the violin plots and how they should be read. On page 5, the first peak we have is in meters (10-100 meters that correspond to 0.01-0.1 kilometers) which has a different measurement unit from the second peak. We’ve added the corresponding value in kilometers for the first peak to prevent the confusion.

 8.    Was any information gathered about the reasons for multiday trips? Besides being longer (and presumably further) were the activities engaged in similar or different to the single day trips? What implications do multiday trips have for understanding the potential for infectious disease transmission?

 Trip information was recorded during the monthly meeting with the field managers regardless of the duration of the trip. Multiday trip may have implications on infectious disease transmission depending on what activities were performed and the location where the participant spent the night. There’s a section later on about consecutive nights spent at farms/forests which is a subset of this multiday trip metric and have more relevance for disease transmission. 9.    Some additional discussion of the reasons for the movement patterns that were observed should be added to the discussion section of the paper. For example, in the results section it is noted that spending more time at the farms usually meant less time in the forests (p. 5) and it is also noted that 20-40 year olds spend the most time in the forest (p. 6). Why is this? How does it relate to the potential for disease transmission?

 We have added/expanded the discussion points in the relevant sections such as the following:  “We surmise that the males of 20-40, most likely being the breadwinners of the family, are subject to any possible subsistence activities and are regarded as the most suitable persons to venture into the forests overnight despite the danger from the wildlife and the harsh living conditions.” 10.    Explain why the pictures in Figure 2 and Figure S6 differ from each other, since both are measuring the utilization of different places. We have explained it in the last paragraph of the discussion section: “Finally, the estimation of land utilization regardless of the method used is imperfect. Having two consecutive GPS points to constitute usage of the land area provide too crude a result (Figure 2). While BRB method provide more accurate and precise estimates (Extended data: Figure S6), it is not without its caveats. BRB assumed that consecutive points that were more than three hours apart were uncorrelated. Since the GPS logger went into sleep mode while stationary, the current land utilization estimation under-estimates the time spent motionless (e.g., sleeping) and hence resulting in lower usage of home in Extended data: Figure S6 compared to that in Figure 2.”

 11.    The discussions of the implications of the observed movement patterns for transmission of diseases prevalent in the study environment needs to be fleshed out. Although this paper is designed to present the results derived from the GPS loggers, there should be a full discussion of what the preliminary data from this study might suggest about how the mobility in the region directly affects disease transmission. Assuming the authors can use their results to derive hypotheses to test in future studies, the discussion is a good place to present those ideas.

 We have added a few discussion points on this.   “There were differences in these movement patterns by demography, with under-20s staying close to home on the majority of the days and both 20-40 and over-40 age groups tending to move farther away each day. We hypothesize that the reason for this difference is that over-20 age groups are more heavily involved in subsistence activities (e.g., farming and foraging which are conducted further away from home) than the under-20 age group.” “Farming is one of the major forms of subsistence for rural families and it must be regarded as relatively safe compared to subsistence activities in the forests that all age groups partake in it.” “We surmise that the males of 20-40, most likely being the breadwinners of the family, are subject to any possible subsistence activities and are regarded as the most suitable persons to venture into the forests overnight despite the danger from the wildlife and the harsh living conditions.” “Spending several consecutive nights in the farms and forests may increase the chances of vector-borne diseases such as malaria Since major malaria vectors in the area such as Anopheles dirus, and Anopheles minimus are found in the deep forests, forest edges, plantations and even in the rice fields. Studies have found that the increased risk of malaria in forest-goers is contributed by inconsistent bed net usage, misconception that alcohol consumption or blankets provides protection against mosquito bites, non-participation in the malaria prevention activities held at the villages.” 

 12.    The paper mentions the importance of the data for “calibrat[ing] human movement patterns in models of infectious diseases”, but there is no discussion at all of what such models are like, what kinds of data are needed in such models, and how the data from the GPS loggers can help calibrate those models. There should either be a substantive discussion of this issue or the use of the data for modeling disease spread should be omitted from the paper at this time.

 We have added a few discussion points on this. “Results from this study, particularly the space utilization data, would be useful in spatially explicit individual-based infectious disease model such as [Gao et. al. 2020] which models the malaria elimination in the rural South East Asian region. Human mobility is a crucial part of many disease transmission dynamics, yet it has been ignored in many infectious disease models because of constraints on data and computational capacity. Compartmental models assume homogeneous mixing of individuals in their respective compartments. While they are quick to set up, they are not suitable for the disease elimination settings. Their homogeneous nature limits the modelers from exploring the impact of multiple interventions tailored towards different risk groups such as forest-goers in malaria intervention. Individual-based models could have individual specific properties and their related movement patterns thus achieving a heterogeneous population. Calibrating on the space utilization data of this study, such models could become more realistic in terms of transmission dynamics. They could provide more accurate and precise estimations to tackle infectious diseases cost-effectively.”

 13.    Finally, on p. 9 the authors mention some limitations related to occupation. However nowhere previous to this in the paper has there been any discussion of occupation. A discussion of the normal activities/occupations of the participants should be included in the introductory material suggested in #1 above. Then, the discussion of occupation as a limitation of the pilot study should be elaborated so it is clear why this is an important issue.    Thank you for this important point/question. We’ve added some text to address this:  “The most commonly reported occupation was farming and most people in this study area, indeed, farm for at least part of the year. However, people in the study area usually perform different types of work according to the season and assigning a single occupation to a person may not be appropriate. Employment in this region is almost entirely informal, and most working-age men will work in agriculture for part of the year and in other types of labor during other parts of the year. Responses to surveys about employment will therefore vary by the time of year, even within a single research participant.”

# Reviewer report: Reviewer response for version 1

**DOI:** 10.21956/wellcomeopenres.18514.r44413

**Published:** 2021-07-16

**Authors:** Steven Stoddard

**Affiliations:** 1 Graduate School of Public Health, San Diego State University, San Diego, CA, USA

The manuscript presents data from a pilot study of human mobility, measured using GPS, for a rural population in Myanmar. The data are interesting, but are too limited to support much analysis that would be of general value (a mistake made in the manuscript). True, mobility patterns are relevant for infectious disease transmission—and many things—but the context provided in the manuscript is too broad to understand the design of the study and justify how movements were sampled. I do think there is great value in exploring these data and thinking about what they suggest about certain movements, but more specificity about the objectives and description of the cultural and environmental context would help understand the data and the importance of collecting them.

 There are too many aspects of ‘human movement’ and not all of them can be perfectly observed, there is always a tradeoff. Even focusing on infectious diseases, there are too many ways different types of movement play a role in transmission, depending on the system. You implicitly made assumptions about which movements were of interest and didn’t justify these. There is theory that provides direction as to which types of movement might matter when, and it would be helpful to this analysis to refer to that theory.

 Altogether, I see value here but recommend revising the introduction to clarify and justify your assumptions, reconsidering some analyses and figures, better describing uncertainty in your data, and editing your discussion and conclusions accordingly. 

- Consider discussing Prothero’s work in your lit review, which did focus on movements in rural communities like yours. I’m not sure the gap is that movements in rural areas are understudied, as you claim, but really it is that we still don’t have a full understanding of the nature and diversity of movement behavior across landscapes and populations, nor how these play into disease transmission. Certainly, the cell-phone data papers have fallen into many of the high-impact journals of late (and your review seems biased toward them), but you don’t do enough of a review to make this claim. But you don’t need to, as the point of the paper is to describe seasonal movements of a rural population using GPS.
- What is the scale of relevant landscape features? How far apart are homes, for instance? For a person who is just stepping out of the house and undergoing regular daily activities, such as going to a school or a shop, how far would they go and how long would it take? Same for other features that people are moving to/from. Put another way, the layout of the landscape will shape the movement kernel, both distance traveled and time spent in residence and in transit.
- Consider trying to describe your data in terms of a movement kernel. This is particularly useful for infectious disease modeling. Basically, you would construct a probability distribution of stays by distance and time spent on a patch. Though your sample is limited, you then have an idea of the probability an individual will move X distance and stay there for dT, which are the metrics one can use to estimate exposure across a population. Your analyses do touch on this idea, but as presented it would be challenging to extract the probability from your paper.
- Please describe the composition of the villages. What types of structures are there? Where do people stay and what are they like? What are the roof materials?
- Please describe the community. Who are the people and what are their lifestyles and livelihoods? What do they farm? Why are they going to the forest? What illnesses are relevant in this area? Malaria? Arboviruses? Why do you think movement will change seasonally?
- Why did you focus on farms, forests, and “homes” (presumably homes were always in a village)?
- How many villages were included?
- How far apart were the clinics? Is there anything different about the catchments they serve? Did you look to see if there was movement variation across clinics? i.e., did the population using clinic 1 move differently than the population using clinic 2?
- When people stay at farms or in the forest, where do they stay? Where do they sleep? Would they be at higher risk to vector borne illnesses? A little more information on the local ecology would be good. The forest isn’t necessarily the high risk area for eg. malaria, the farms might be. But if the villages are small and you have more anthropophilic mosquitoes like P falciparum, the villages may be the risk area. And conversely, respiratory illnesses will be more of a risk in the village.
- It would help the paper if you defined what epidemiological risk you are interested in. What motivated the study? Surely not just any infectious disease, because you made specific sampling choices that may or may not be as informative for directly transmitted or other pathogens.
- Was monitoring continuous over the time of participation? Does figure S3 show the time that participants carried the devices or the time that the devices were actually recording data? You say the charge was roughly 1-1.5 months and you rotated monthly. I hardly expect they all worked perfectly. Please say something about monitoring fidelity, i.e., proportion of the observation period over which you actually have tracks.
- By sampling every 30 min, you are choosing to sample movements that are longer in time (and often distance). Why? Understandably this extends the sampling period because the devices’ battery charge lasts longer, but you miss many small-scale movements that will be highly relevant for most pathogen transmission events. On the upside, you do a better job of sampling day-to-day variability and capture more longer distance movements. Please provide justification for your sampling focus. Note that stopping the tracking at 6 pm also cuts out many potential relevant movements at a time when many vectors are active.
- Note also that letting the devices ‘go to sleep’ after being still for an hour inevitably leads to missed points, especially when these stays are in indoor locations. You didn’t do any validation of your GPS tracking, so it is worth discussing.
- I’d like to see more discussion of your definition of ‘home’ and whether your approach was valid. Given the uncertainty in any single GPS reading, I don’t see how you can be sure that the last point of the day - at 6 pm no less (especially without some explanation for why people would not move at all after this time) - is the point indicating where they stayed the night. The accuracy of single GPS readings is variable and tends to be worse in and around structures but much better when outside and in movement. So identifying ’stays’ would require a ‘cloud’ of points indicating that a specific site is repeatedly visited and used. Also, a radius of 266 m for a home buffer seems quite large. What fits within that radius? Aren’t these small villages? What is the scale of the villages? I would think you would know the location of the home a priori based on recruitment and then could better define a person’s immediate ‘home range’ based on point density (with the caveat that your weren’t really sampling frequently enough to capture fine scale movement).
- Why Euclidean distance? Is ‘as the crow flies’ really the most parsimonious approach or just the easiest? Did you consider other options? A friction surface based on landcover and/or DEM, for instance? Or based on roads/tracks?
- Why use a violin plot for Figure 2? Did you intend to show distributions for males and females? Otherwise, why not just overlay them so the discrepancies could be more clear?
- What were the actual ages of the <20? Your methods state you ‘targeted adults’. What ages? 18 and up? 16 and up?
- Why do you break down your age data into the bins you selected? Your data are really quite limited, it might be better to not bother at all.
- How did movement vary across individuals originating in different villages?
- Figure 3 A is illegible when printed.
- I’m not sure what you are trying to do with figure 5 and recommend you either remove it or reconceive. It would be useful to see the distribution of residence time, but boxplots or similar might be more illustrative.
- pg 6 you state “the negative binomial regression provided strong evidence…” Not sure this can be the case, especially since you have a convenience sample that by your admission was short for females. You can’t make any inferences with these data for this reason, so should stick to descriptive statistics or otherwise be careful about how you use any models.

Is the work clearly and accurately presented and does it cite the current literature?

Yes

If applicable, is the statistical analysis and its interpretation appropriate?

Partly

Are all the source data underlying the results available to ensure full reproducibility?

Partly

Is the study design appropriate and is the work technically sound?

Yes

Are the conclusions drawn adequately supported by the results?

Partly

Are sufficient details of methods and analysis provided to allow replication by others?

Yes

Reviewer Expertise:

infectious disease epidemiology and ecology; human mobility and disease transmission

I confirm that I have read this submission and believe that I have an appropriate level of expertise to confirm that it is of an acceptable scientific standard, however I have significant reservations, as outlined above.

# Response

**Published:** 2023-10-23

**Authors:** Daniel Parker

**Affiliations:** University of California, Irvine, USA

Dear Editors and Reviewers; Thank you for your helpful comments and suggestions. We would also like to take this opportunity to apologize for our slow movement on addressing your comments and critiques. In the last few years there has been a military coup in Myanmar, on top of the extreme challenges that the pandemic of SARS-CoV-2 has presented, and this has delayed us in our focus on this manuscript and in many other ways. We have also been experiencing some debilitating health issues that have slowed our work. We very much appreciate the   time and effort you put into reviewing our manuscript and we hope that we’ve made it clear that our delays in addressing these comments are because of external factors.

 We have attempted to address each of your comments and suggestions and we believe that the manuscript is now much improved. Please see our line-by-line responses below:

 Reviwer 1: Steven Stoddard, Graduate School of Public Health, San Diego State University, San Diego, CA, USA

 The manuscript presents data from a pilot study of human mobility, measured using GPS, for a rural population in Myanmar. The data are interesting, but are too limited to support much analysis that would be of general value (a mistake made in the manuscript). True, mobility patterns are relevant for infectious disease transmission—and many things—but the context provided in the manuscript is too broad to understand the design of the study and justify how movements were sampled. I do think there is great value in exploring these data and thinking about what they suggest about certain movements, but more specificity about the objectives and description of the cultural and environmental context would help understand the data and the importance of collecting them.

 There are too many aspects of ‘human movement’ and not all of them can be perfectly observed, there is always a tradeoff. Even focusing on infectious diseases, there are too many ways different types of movement play a role in transmission, depending on the system. You implicitly made assumptions about which movements were of interest and didn’t justify these. There is theory that provides direction as to which types of movement might matter when, and it would be helpful to this analysis to refer to that theory.

 Altogether, I see value here but recommend revising the introduction to clarify and justify your assumptions, reconsidering some analyses and figures, better describing uncertainty in your data, and editing your discussion and conclusions accordingly. 

 •    Consider discussing Prothero’s work in your lit review, which did focus on movements in rural communities like yours. I’m not sure the gap is that movements in rural areas are understudied, as you claim, but really it is that we still don’t have a full understanding of the nature and diversity of movement behavior across landscapes and populations, nor how these play into disease transmission. Certainly, the cell-phone data papers have fallen into many of the high-impact journals of late (and your review seems biased toward them), but you don’t do enough of a review to make this claim. But you don’t need to, as the point of the paper is to describe seasonal movements of a rural population using GPS.

 Thank you for your suggestions. We have included some brief discussion about Prothero’s work in our revised manuscript and have dropped the statement about empirical movement studies being relatively rare in resource poor settings. 

 •    What is the scale of relevant landscape features ? How far apart are homes, for instance? For a person who is just stepping out of the house and undergoing regular daily activities, such as going to a school or a shop, how far would they go and how long would it take? Same for other features that people are moving to/from. Put another way, the layout of the landscape will shape the movement kernel, both distance traveled and time spent in residence and in transit.

 Thanks for these important comments and points. Landscape features, including house locations, are certainly important in assessing movement processes. In this study we did not recruit from villages or houses, but rather from clinics located on the Thai side of the international border. We don’t have GPS points per house. From our experience working in this setting, we know that these types of features vary quite a lot from village to village – and so it really isn’t possible to say how far someone would normally have to travel to go to a school or shop. For example, relatively few villages will have a school, even fewer would have a secondary school, etc. Indeed, one of the major goals of doing this type of work in this setting would be to develop an understanding  the size and shape of the movement kernel.

 •    Consider trying to describe your data in terms of a movement kernel  . This is particularly useful for infectious disease modeling. Basically, you would construct a probability distribution of stays by distance and time spent on a patch. Though your sample is limited, you then have an idea of the probability an individual will move X distance and stay there for dT, which are the metrics one can use to estimate exposure across a population. Your analyses do touch on this idea, but as presented it would be challenging to extract the probability from your paper.

 Thanks for this suggestion. This seems very similar to the approach we took with the biased random bridges, with kernels shown for one individual in Figure 3B. Each individual has a different utilization probability, so we aren’t sure that providing plots for all would be optimal for the paper. If researchers would like to work with these data, we are open to that possibility (see data access statement) but we aren’t sure that there is an appropriate way to accurately provide such information in the manuscript. We would essentially need 50+ probability distributions and readers would still need to rely on interpreting the plots to estimate the different probability distributions (with multiple kernels per research participant).  

 •    Please describe the composition of the villages. What types of structures are there? Where do people stay and what are they like? What are the roof materials?

 We have provided some text about normal household structures now, but this was not a village or house-based study so we do not have information about participant households. We have attempted to make our recruitment process more clear in the text now. Please see the section on the “Context of the study area” in the manuscript and copied underneath.

 “Context of the study area The study area is on the Thailand-Myanmar border. Participants were recruited from clinics that serve rural, mostly underdeveloped, and low population density communities. Most participants were of the Karen ethnic group. Villages were made up of a few dozen of mostly multigeneration families living in stilt houses made of wood and thatched roof. Villages didn’t normally have schools, clinics, or sanatory toilets. The houses are normally located along the main dirt road of the village. The dirt roads then continued to connect to other villages and small towns through a hilly and rugged terrain with occasional watersheds and river basins which made traversing difficult, especially during the rainy season.  Villagers made their living mostly though agriculture, but they have to undertake various types of jobs throughout the year for their subsistence. They developed land in and around their villages into farms to cultivate rice and vegetables. They farm poultry and pigs under their stilt houses. Some villagers go into the forests for hunting, or for foraging wood, and to collect wild edibles. They would go to the farms and forests overnight occasionally, and sleep without much protection from mosquitos and insects. We focused on farms and forests as places of interest in this study since apart from their homes, farms and forests might be the places the villagers spend significant amount of their time while being vulnerable to infectious diseases such as malaria.” •    Please describe the community. Who are the people and what are their lifestyles and livelihoods? What do they farm? Why are they going to the forest? What illnesses are relevant in this area? Malaria? Arboviruses? Why do you think movement will change seasonally?

 Please see the section on the “Context of the study area” in the manuscript. 

 •    Why did you focus on farms, forests, and “homes” (presumably homes were always in a village)?

 One motivating factor for this work is that the ecology of malaria in this setting remains poorly understood. There is quite a bit of literature about ‘forest malaria’ – even going back to Prothero – with relatively scant data about the amount of time spent in forests. Most of the people living in this setting are farmers for at least part of the year. We therefore focus on villages, farms (which are normally near villages), and forests (which tend to be on the outskirts of farms) as these tend to be the places where folks spend most of their time and since forests have long been suspected as important places for Plasmodium falciparum transmission. 

 Home location in the study is derived from the median center of all the GPS points where an individual spent the night, which in turn is derived from the last GPS point of the day between 6pm to 12 midnight.

 •     How many villages were included?  We have added the fact that 10 villages were included in the study: 

 “Participants were recruited from 10 villages (the lowest level of administrative division in Myanmar) near two clinics on the Thailand-Myanmar border: Wang Pah and Maw Ker Tai Clinics.”

 •    How far apart were the clinics? Is there anything different about the catchments they serve?  Did you look to see if there was movement variation across clinics? i.e., did the population using clinic 1 move differently than the population using clinic 2?

 The clinics are approximately 57km apart from each other using a straight line distance. There are some environmental differences between the catchment areas. The southern clinic is nearer to the mountain range and forests/jungles whereas the northern clinic is surrounded by hilly agricultural fields. We did include the recruitment place in the generalized linear mixed effect model. We dropped the variable because it didn’t improve the model.  

 •    When people stay at farms or in the forest, where do they stay? Where do they sleep? Would they be at higher risk to vector borne illnesses? A little more information on the local ecology would be good. The forest isn’t necessarily the high risk area for eg. malaria, the farms might be. But if the villages are small and you have more anthropophilic mosquitoes like P falciparum, the villages may be the risk area. And conversely, respiratory illnesses will be more of a risk in the village.

 Thank you for this important comment/question. More information on this have been added as a context of the study area: “Villagers made their living mostly though agriculture, but they have to undertake various types of jobs throughout the year for their subsistence. They developed land in and around their villages into farms to cultivate rice and vegetables. They farm poultry and pigs under their stilt houses. Some villagers go into the forests for hunting, or for foraging wood, and to collect wild edibles. They would go to the farms and forests overnight occasionally, and sleep without much protection from mosquitos and insects. We focused on farms and forests as places of interest in this study since apart from their homes, farms and forests might be the places the villagers spend significant amount of their time while being vulnerable to infectious diseases such as malaria.”

 •    It would help the paper if you defined what epidemiological risk you are interested in. What motivated the study? Surely not just any infectious disease, because you made specific sampling choices that may or may not be as informative for directly transmitted or other pathogens.

 The study was not designed to estimate epidemiological risk for any specific disease. It was a pilot study to see if it would be possible to use GPS loggers to study human movement patterns in this setting – which is notable because this is a difficult setting to work in because of the political and civil conflict situation, both historically and in the present. A potential next step could be to assess risk of malaria infection, and in some ways this motivated the present study – but the present study was not designed to specifically study risk of malaria infection. Results from studies like this should be relevant for other environmentally transmitted diseases as well (especially vector-borne diseases).  

 •    Was monitoring continuous over the time of participation? Does figure S3 show the time that participants carried the devices or the time that the devices were actually recording data? You say the charge was roughly 1-1.5 months and you rotated monthly. I hardly expect they all worked perfectly. Please say something about monitoring fidelity, i.e., proportion of the observation period over which you actually have tracks.    

 Yes, monitoring was continuous over the time period. Figure S3 only showed the first and the last time stamp of the GPS device that the participant carried.  Unless the battery of the GPS device ran out, or the participant left the device forgotten somewhere, the monitoring was continuous. We had one technical difficulties with only 1 device in the study, which was replaced by a working device in the next 1-1.5 month interval. The mean percentage of days GPS points were actually observed for each participant over the study is 86.17% (median: 91.21, IQR: 79.54 to 96.93).

 •    By sampling every 30 min, you are choosing to sample movements that are longer in time (and often distance). Why?  Understandably this extends the sampling period because the devices’ battery charge lasts longer, but you miss many small-scale movements that will be highly relevant for most pathogen transmission events. On the upside, you do a better job of sampling day-to-day variability and capture more longer distance movements. Please provide justification for your sampling focus. Note that stopping the tracking at 6 pm also cuts out many potential relevant movements at a time when many vectors are active.

 The primary goal of this study was to assess the feasibility of doing GPS logger work in this setting. We also wanted to see if it would be possible to assess seasonal variations in movement behavior (i.e. could a longitudinal GPS logger study on human movement patterns work in this setting?) We chose 30 minute intervals as a balance between capturing short-term movements and having data over a long period of time, with a need to keep interview/visits to a minimum because of funding and labor constraints. Shorter time intervals would have meant either more frequent visits with the research participants to replace devices with fully charged ones, or we would have had to significantly shorten the study period (losing the longitudinal nature of the data collection).  Also, the GPS tracking doesn’t stop at 6pm.  GPS points recorded between 6pm to 12 midnight were used to estimate the home location of the participant or the location where the participant spent the night.

 •    Note also that letting the devices ‘go to sleep’ after being still for an hour inevitably leads to missed points, especially when these stays are in indoor locations. You didn’t do any validation of your GPS tracking, so it is worth discussing.

 The devices ‘awaken’ when the devices are moved so that dormant GPS devices do restart recoding upon detection of movement. “Figure S2: GPS reading errors in stationary devices.” discusses the error compared to the actual GPS location under different scenarios. 

 •    I’d like to see more discussion of your definition of ‘home’ and whether your approach was valid. Given the uncertainty in any single GPS reading, I don’t see how you can be sure that the last point of the day - at 6 pm no less (especially without some explanation for why people would not move at all after this time) - is the point indicating where they stayed the night. The accuracy of single GPS readings is variable and tends to be worse in and around structures but much better when outside and in movement. So identifying ’stays’ would require a ‘cloud’ of points indicating that a specific site is repeatedly visited and used. Also, a radius of 266 m for a home buffer seems quite large. What fits within that radius? Aren’t these small villages? What is the scale of the villages? I would think you would know the location of the home a priori based on recruitment   and then could better define a person’s immediate ‘home range’ based on point density (with the caveat that your weren’t really sampling frequently enough to capture fine scale movement).

 The GPS tracking didn’t stop at 6pm. The last GPS point of the day is actually selected from the timeframe between 6pm to 12 midnight. We agree that there’s a ‘cloud’ of points around a site that is visited frequently (Figure S2). Participant’s home location (a single point) is derived from the median center of the cloud of all the GPS points where s/he spent the night, each of which in turn is derived from the last GPS point of the day between 6pm to 12 midnight. The 266 meters radius buffer comes from the standard deviation from the median centre for the worst performing test (in a bag, inside a house) as explained in Figure S2: GPS reading errors in stationary devices. It is a conservative estimate to judge whether or not a participant’s movements were likely real or the result of measurement error. The buffer usually only covers a section of the village.

 •    Why Euclidean distance? Is ‘as the crow flies’ really the most parsimonious approach or just the easiest? Did you consider other options? A friction surface based on landcover and/or DEM, for instance? Or based on roads/tracks?  In some ways, Euclidian distance is the most parsimonious for this setting for a variety of reasons, even though it is an oversimplification of how actual movements would work. A friction surface would be more sophisticated, but would inherently rely on assumptions that make even less sense in this setting than in others. This setting is an international border in a conflict setting. Movements that might make sense from a ease of travel perspective often don’t align with what is safe. Roads are often lined with check points, for example, and so while they may seem to be the most logical travel route many people will actually avoid them. Using Euclidian distances also allows for calculating distances that are intuitive for comparing across individuals or demographic groups. 

 •    Why use a violin plot for Figure 2? Did you intend to show distributions for males and females? Otherwise, why not just overlay them so the discrepancies could be more clear?   Violin plot is used in Figure 1 to show the location of the peaks together with the boxplot, as well as whether or not the distributions are statistically different at the 5% level between the three age groups. In this case, all three groups have two peaks, but the one in <20 age group is different from the other two (P <= 0.0001) since its first peak is much higher/broader than the other two. We believe that the difference could still be discerned from the current violin plot. 

 •    What were the actual ages of the <20? Your methods state you ‘targeted adults’. What ages? 18 and up? 16 and up?    Yes, we targeted individuals who were 18 years of age and above. We have now added this into the text: “The study targeted individuals who were 18 years of age and above adults from the Karen or Burmese ethnic groups, who stated that they would be able to keep track of the GPS device, who were capable of walking outside of village boundaries at recruitment, and who were willing to provide written consent to the study.”

 •    Why do you break down your age data into the bins you selected? Your data are really quite limited, it might be better to not bother at all. We use the bins largely because of the small sample sizes. Most of the analyses and plots would not be possible with age as a continuous variable, especially with such a small cohort. Using age bins is also common practice in both epidemiology and demography, partially for dealing with problems around age-heaping. It is even more important in smaller sample sizes. 

 •    How did movement vary across individuals originating in different villages? Since we did not recruit by village, we did not look at potential variations between villages and since the cohort size is quite small (roughly 50 participants from 10 villages) comparisons aren’t really possible between villages. 

 •    Figure 3 A is illegible when printed. We have now increased the font size. The new figure is linked here.

      

 •    I’m not sure what you are trying to do with figure 5 and recommend you either remove it or reconceive. It would be useful to see the distribution of residence time, but boxplots or similar might be more illustrative. Fig. 5 is shows how certain age group (20-40) of males consistently frequent the forest or stay the longest time in the forest. Boxplots alone would not be able to describe the age group and gender. We would prefer to keep the figure in the text.

 •    pg 6 you state “the negative binomial regression provided strong evidence…” Not sure this can be the case, especially since you have a convenience sample that by your admission was short for females. You can’t make any inferences with these data for this reason, so should stick to descriptive statistics or otherwise be careful about how you use any models.   We agree that caution should be used in interpreting the results from this analysis, in large part because of the convenience sample. We do believe that this analysis is applicable to the cohort though. We have added some text to soften this statement:  “The negative binomial regression provided strong evidence that males in this cohort were more likely to spend nights in farms (p=0.045) and in forests (p=0.01) compared to females, and that young adults (the 20-40 age group) were more likely to spend nights in the forest compared to the under-20 age group (p=0.043), after controlling for the remaining variables (Table 2)."
